# Supplementary material for: The views of public and clinician stakeholders on risk assessment tools for post-stroke dementia: a qualitative study
Source: BMJ Open. 2019 Mar 27;9(3):e025586. doi: 10.1136/bmjopen-2018-025586 (PMC6475139; doi:10.1136/bmjopen-2018-025586)
Supplement: Author's manuscript [file bmjopen-2018-025586.draft_revisions.pdf]

BMJ Open is committed to open peer review. As part of this commitment we make the peer review history of every article we publish publicly available.

When an article is published we post the peer reviewers' comments and the authors' responses online. We also post the versions of the paper that were used during peer review. These are the versions that the peer review comments apply to.

The versions of the paper that follow are the versions that were submitted during the peer review process. They are not the versions of record or the final published versions. They should not be cited or distributed as the published version of this manuscript.

BMJ Open is an open access journal and the full, final, typeset and author-corrected version of record of the manuscript is available on our site with no access controls, subscription charges or pay-per-view fees (http://bmjopen.bmj.com).

If you have any questions on BMJ Open's open peer review process please email info.bmjopen@bmj.com

BMJ Open

The Views of Public and Clinician Stakeholders on Risk Assessment Tools for Post-Stroke Dementia: A Qualitative Study

Journal:	*BMJ Open*
Manuscript ID	bmjopen-2018-025586
Article Type:	Research
Date Submitted by the Author:	23-Jul-2018
Complete List of Authors:	Tang, Eugene; Newcastle University, Institute of Health and Society<br>Exley, Catherine; Northumbria University, Faculty of Health and Life Sciences<br>Price, Christopher; Newcastle University, Institute of Neuroscience, Stroke Research Group<br>Stephan, Blossom; Newcastle University, Institute of Health and Society<br>Robinson, Louise; Newcastle University, Institute of Health and Society
Keywords:	PRIMARY CARE, STROKE MEDICINE, QUALITATIVE RESEARCH, Dementia < NEUROLOGY

SCHOLARONE™ Manuscripts

For peer review only

The Views of Public and Clinician Stakeholders on Risk Assessment Tools for Post-Stroke Dementia: A Qualitative Study

Dr. Eugene Yee Hing Tang[1,2] e.y.h.tang@newcastle.ac.uk

Prof. Catherine Exley[3] catherine.exley@northumbria.ac.uk

Dr. Christopher Price[4] c.i.m.price@newcastle.ac.uk

Dr. Blossom CM Stephan[1,2] blossom.stephan@newcastle.ac.uk

Prof. Louise Robinson[1,2] a.l.robinson@newcastle.ac.uk

[1] Institute of Health and Society, Newcastle University, Baddiley-Clark, Richardson Road, Newcastle upon Tyne, UK, NE2 4AX

[2] Newcastle University Institute of Ageing, Newcastle University, Campus for Ageing and Vitality, Newcastle upon Tyne, UK, NE4 5PL

[3] Faculty of Health & Life Sciences, Northumberland Building, Northumbria University, Newcastle upon Tyne, NE1 8ST

[4] Institute of Neuroscience, Stroke Research Group, Newcastle University

Corresponding author: Dr. Eugene Yee Hing Tang, Institute of Health & Society, Newcastle University, Level 2, Newcastle Biomedical Research Building, Campus for Ageing and Vitality, Newcastle upon Tyne, NE4 5PL, T: 0191 208 8758,

E: e.y.h.tang@newcastle.ac.uk

For peer review only

Abstract

OBJECTIVE: Stroke-survivors are at increased risk of future dementia. Assessment to identify those at high risk of developing a disease using predictive scores has been utilised in different areas of medicine. A number of risk assessment tools for dementia have been developed but none has been recommended for use clinically. The aim of this qualitative study was to assess the acceptability and feasibility of using a risk model to predict post-stroke dementia.

DESIGN: Qualitative semi-structured interviews were conducted with thematic analysis. Patients and carers were also offered a follow-up interview at 12 months.

SETTING: The study was conducted in the North-East of England with stroke patients, family carers and healthcare professionals in primary and secondary care.

PARTICIPANTS: Thirty-nine (17 clinicians and 15 stroke patients and their carers at baseline. Twelve stroke patients and their carers were also seen at follow-up) interviews were conducted.

RESULTS: Barriers and facilitators to risk assessment were discussed by each group. For patients and carers the focus for facilitators were based on the outcomes of risk assessment i.e. an assessment could assist with preparation, diagnosis and for reassurance. For clinicians, facilitators were focused on the process i.e. familiarity in primary care, resource availability in secondary care and collaborative care. For barriers, both groups focussed on the outcome including for example the anxiety generated from a potential diagnosis of dementia. For patients and carers a further barrier included concerns about how it may affect their recovery. For clinicians there were concerns about limited interventions and how it would be different from standard care.

For peer review only

CONCLUSIONS: Risk assessment for dementia after stroke presents challenges given the ramifications of a potential diagnosis of dementia. Attention needs to be given to how information is communicated, and the strategies developed to support patients and carers if risk assessment has taken place.

For peer review only

Strengths and Limitations of the Study:

- To the best of our knowledge this is the first qualitative study to examine critically the views of stroke patients and their family carers and clinicians about the acceptability and feasibility of a risk assessment approach to assist in earlier identification of post-stroke dementia.
- Understanding stakeholder views on risk assessment for dementia can help inform future strategies if risk assessment for dementia is used to assist with earlier diagnosis.
- Patient participants came from one area of England who were able to attend hospital outpatient departments and so may not represent the views and experiences of those with more severe post-stroke sequelae.
- Clinician participants came from one area of England and so may not represent the views of other service models in other regions of the UK.
- It is recognized that clinicians tended to be more familiar with the process of risk assessment and could elaborate further on the process involved.

For peer review only

Introduction

There is currently no cure for dementia and it is estimated that the worldwide economic burden will rise to US$2 trillion by 2030[1]. It has been suggested that the most powerful way to affect costs is by reducing the numbers of people who develop the illness. This may be facilitated by prediction of individual risk for the disease. Stroke is associated with an increased risk of dementia and cognitive impairment[2-4]. A history of stroke doubles the risk of incident dementia in older populations, a risk that is independent of demographic and cardiovascular risk factors and prestroke cognitive decline[2]. Stroke incidence and numbers of stroke-survivors are likely to increase due to simultaneous ageing populations and declining stroke mortality rates[5]. Given that the incidence of dementia increases exponentially with age[1 6], this will mean that post-stroke dementia will also become increasingly prevalent. It will therefore be important to identify those at greatest risk of developing dementia following stroke in order to implement strategies to reduce risk.

Risk prediction models for dementia to identify those at higher risk have been developed in whole populations[7 8] with some models specifically developed to predict cognitive impairment and dementia in stroke populations[9-12]. These stroke-specific models predict dementia or cognitive impairment over a relatively short time period. In spite of the expanding research in this field, none of the dementia risk prediction tools have been clinically implemented. Further, no studies have assessed the feasibility or acceptability of implementing such a strategy in a stroke population. Although risk models are currently used in everyday clinical practice in other branches of medicine, in particular prevention of cardiovascular[13] and cerebrovascular[14] disease, it is unclear how clinicians would feel about using a similar strategy to predict dementia. Further, no studies have evaluated whether

For peer review only

using risk assessment tools for dementia would be acceptable to stroke patients themselves.

This paper presents findings from a qualitative study conducted with patients, carers and clinicians, which sought to critically examine their views about the acceptability and feasibility of using risk prediction models in post-stroke care to identify those at greatest risk of future dementia.

For peer review only

Methods

Patient and Public Involvement

Patients and members of the public have been involved in the development of this study from the beginning of the proposal. A participant advisory group also oversees the work conducted and annual face-to-face meetings are held to inform them of the study findings. The participant advisory group consists of members from a stroke research patient and carer panel, an organisation aimed at capturing public views about research and from a dementia and neurodegeneration specialty PPI group. The same group reviewed the study materials to ensure suitability particularly for stroke-survivors and their family carers.

Ethical Approval

The study was conducted in the North East of England. Ethical Approval was obtained from the London – Hampstead Research Ethics Committee (reference 16/LO/0133). Participants provided informed written consent prior to the interview.

Data collection

Interviews were conducted between April 2016 and July 2017 by one researcher (EYHT). The topic guide was initially derived from relevant literature and expert

clinical views within the research team. It was designed to be iterative to enable any topics, which had not been previously identified, to be pursued in subsequent interviews. Face to face semi-structured interviews were conducted with all but one participant (clinician) who had a telephone interview. The patient and family carer were interviewed individually or in pairs as requested by participants. Clinicians were interviewed individually. The interviews focussed on the benefits and challenges of improving earlier diagnosis of dementia after stroke. This included specific questions on the delivery of this assessment (e.g. who should carry it out), what variables could be used and how best to manage the outcome. The interviews also sought the views of stakeholders on the care experience of post-stroke individuals with memory problems. The views of clinicians have been reported elsewhere[15] and the views of patients and carers will be reported separately. The process of risk assessment was described to participants. This was further emphasised with examples of published tools in order to highlight examples of variables used to ensure participant understanding of the process. Informed written consent was obtained from all participants prior to the interview commencing. All interviews were audio-recorded and then transcribed verbatim. To protect participant anonymity, unique identifiers were used throughout the process with identifiable personal data removed before analysis was conducted.

Patient and Carer Sampling

Patients and carers were purposively sampled from stroke clinics. As part of routine clinical practice in United Kingdom (UK) stroke services, all stroke-survivors are invited to a specialist review at six months after the event which includes a general enquiry about memory concerns[16]. If the patient reported any subjective memory concerns at the clinic and was over the age of 60, the stroke specialist nurse would

For peer review only

provide further study information. Family carers were also recruited if they were involved in the stroke-survivor's care, for example, if they attended the clinic appointment with them. If potential participants were interested in taking part in the study, their details were passed onto the research team. On receipt of this information the patient or carer was contacted by one researcher (EYHT) to provide detailed information and an opportunity to ask questions before agreeing to participate. Participants were asked to take part in an interview immediately following their six-month review and/or six months later.

Clinician Sampling

General Practitioners (GPs) and secondary care clinicians (stroke consultants and specialist nurses) in the North East of England were contacted to participate in the study. Participants were given an opportunity to ask further questions. Clinicians were purposively sampled to ensure that a broad range of care professionals in both primary and secondary care were recruited.

Data analysis

Interview data was analysed using thematic analysis[17] following the principles of constant comparative methods[18]. One researcher (EYHT) familiarised himself with the dataset and subsequently coded the transcripts line-by-line. Initially, a small subset of transcripts were analysed to identify initial themes and these were discussed between CE and EYHT. Data collection and analysis was iterative and as interviews progressed, further analysis led to new themes emerging and refinement of existing themes and subthemes, which were subsequently grouped into broad categories to facilitate interpretation[19]. The wider team (EYHT, CE, LR, BS and CP) discussed and agreed on the final categories which are presented below. For patient

For peer review only

and carer interviews, where follow-up interview data was also obtained, these were analysed as separate interviews to assess for any change in views over time. There was particular focus to understand what was important to patients, carers and clinicians. Data analysis was facilitated by a data software handing package (NVivo version 11). The paper conforms to the Standards for Reporting Qualitative Research checklist[20] (please see supplementary table 1).

Results

In total, 30 baseline interviews were conducted, analysed and compared including: 15 patient and carer interviews (see table 1) and 17 primary and secondary care clinician interviews (see table 2). Eight stroke-survivors and four carers agreed to a further follow-up interview six months later with nine interviews completed. The data from this study suggest that in terms of risk assessment facilitators and barriers exist to implementation. Whereas patient facilitators focussed on the outcome of the risk assessment, clinicians focussed more on the process of risk assessment for facilitators. Both groups discussed some potential barriers associated with risk assessment focussing on the outcome.

Patient and Carer Views: Facilitators to Risk Assessment Focuses on the Outcome of Assessment

When stroke-survivors and carers discussed the concept of risk assessment, the overarching theme was that an assessment outcome was what was important, irrespective of the process and clinicians involved. Participants focussed on several areas of why the outcome was important to them.

For Preparation

For peer review only

Some stroke-survivors were generally positive about receiving a risk assessment for dementia. They acknowledged that a diagnosis was something that could enable individuals to prepare themselves both at baseline and subsequently at follow-up interview:

"It's the same as knowing and not knowing, if you know that something is approaching. Not everybody is the same with the problem. You might be able to deal with it in a different way or the person supporting you, the nurse or whoever, might be able to find a different way or a more positive way of managing it." (P6, male, stroke-survivor at follow-up interview)

Similarly, for carers, there was the emphasis on what could be done following the assessment. One carer emphasized the importance of looking after the whole person, and, how earlier recognition of a potential dementia diagnosis could ensure strategies were in place to help the individual:

"But I think, if you look at the whole thing of this care of this person, if we knew earlier that you know the chances are that your memory is going to get bad and you are going to go into dementia or whatever, then we can start thinking, "Right, well let's prop it up, let's think of ways in helping your memory as it is, to maintain the level it is before you've got no choice, it's going to get worse." You know, maintaining what you've got and different ways of maintaining it, I think that would help." (C5, female carer (daughter) of stroke-survivor)

For Timely Diagnosis

For some stroke-survivors it did not matter who was performing the risk assessment for dementia or where it was undertaken. What was important was that the diagnosis was reached at the right time:

For peer review only - http://bmjopen.bmj.com/site/about/guidelines.xhtml

"I wouldn't say it matters, as long as it's diagnosed at the right time." (P5, male stroke-survivor)

To enable this, when discussing who should perform the risk assessment, carer participants felt that primary care and the community were regarded as being optimal because of the existing GP-patient relationship, with the GP having an overall view of the individual's care, rather than the focus on the process of evaluation:

"I think if you've got a good relationship with your GP I think it should be that, it should be them. Yeah, because you know you trust them you build up a relationship with them so I think that probably, for me that would be the one." (C4, female carer of stroke-survivor)

For Reassurance

When stroke-survivor participants were asked about a structured risk assessment process, they reported that the outcome could also ensure some reassurance, either that their symptoms were not related to a dementia diagnosis or that a diagnosis of dementia would be accompanied by support information:

*"I think it's reassurance a lot of reassurance with people. You have to give them that [*they are at low risk] *to tell them, that "We are there with you. We're going to be helping you." And thats you know, I think that's a good thing.*" (P2, female stroke-survivor)

Patient and Carer Views: Barriers to Risk Assessment Focuses on the Outcome of Assessment

Anxiety around a potential diagnosis of dementia

For peer review only

Some carers commented on how the outcome from risk assessment could generate worry and anxiety because of the potential diagnosis of dementia:

“To be honest, I don’t know if it would help somebody saying, “You're like this, you’re upset because you're like this now, but we actually think you're going to get much worse.” Do you know what I mean?” (C3, female carer (daughter) of stroke-survivor)

This person’s opinion did not change when she was followed-up six months later. The participant’s focus was again on worrying about what could develop and how ignorance and not knowing about one’s risk would actually be more preferable:

“If you could find out and then say, "Right, we've got this medication, or something, that can help you," maybe. But if they're just going to tell you, and then you've got this hanging over your head, and you're thinking, "When is it going to start?" and then you’d be thinking you'd forget something and you'd think, "Oh, that's it, it's coming", which it would be quite normal if you hadn't had that diagnosis, you'd think, "Well I just forgot something, everybody does that." (C3, female carer (daughter) of stroke-survivor at follow-up interview)

However, one carer felt that despite the worry a potential diagnosis may generate, the benefit of this would be to find strategies to maintain cognitive function:

“I think if you had earlier diagnosis, then you would be sort of prepared before things got difficult to handle, or before problems arise, that would be a very good thing. The disadvantages as you say, alarming the carers or the patients themselves, “I’m going to lose my mind.” Because, particularly in the older generation, that’s a big worry to them. It is a big worry, it’s a big worry to all of us, but to older people particularly.” (C5, female carer (daughter) of stroke-survivor)

Concerns about how it may affect their recovery

For peer review only - http://bmjopen.bmj.com/site/about/guidelines.xhtml

Not all stroke-survivors were as keen to engage in risk assessment, as there was emphasis on how this may affect them psychologically particularly when their physical deficits had recovered enough to allow them to return to a more usual routine. Therefore, although diagnosis was felt to be important, whether an individual would like to know was also dependent on their subsequent post-stroke recovery:

"That's difficult you know because I mean if you have an early diagnosis you know and say, well "It's going to happen" you know but at the moment now I seem to be progressing through, I'm driving now, you know I'm going back to meetings and whatever. I wonder whether an early diagnosis would restrict that." (P4, male stroke-survivor)

This was particularly evident when patients were followed up six-months later. One participant had actually changed her view over time. Although she had initially felt positive about the process, she then changed her mind when questioned on the same process at her follow-up interview:

"I think my thinking has gone the other way for knowing about that. I think it's sad. I think it's a sad thing. I really do, I think it's really sad that for people to know that they're going to be at high risk, it's a sad thing for it to happen to people, and I don't think I'd want to be one of the sad people. I think I'd just want to be, potter along and that's it." (P2, female, stroke-survivor at follow-up interview)

At follow-up interviews participants also felt that risk assessment should be an individual choice because of the ramifications of the assessment outcome i.e. a potential diagnosis of dementia. Although clinicians may deem it to be helpful, the choice to undergo risk assessment needs to be a weighed up, which should negate any calls for it to be made a universally applied process:

“I think, medically speaking, yes. On the other hand, does it give people things to worry about that they wouldn’t have worried about if you hadn’t done the tests? So, I think it depends really on your personal point of view. Do you want to be, you see I would look on the test as saying, well you’re at a low, you’ve got a low risk so that’s great but then if it turned out you’d got a high risk are you going to be more worried and less happy than you were before. It’s hard to really balance it, isn’t it? (P3, female, stroke-survivor at follow-up interview)

Clinician Views: Facilitators to Risk Assessment Focusses on the Process

Clinicians discussed facilitators to risk assessment in terms of how the process may affect the individual and also how the process could be implemented in the future.

When discussing how to implement this process, both primary and secondary care specialists discussed the advantages associated with hosting this process within their own individual teams.

Process familiarity in Primary Care

For primary care, it was about the fact that risk assessment was already a familiar process but that it needed to be individualised:

“I think it's a good tool. We're quite good at using tools, aren't we, but there’s always going to be exceptions to the rules and you've got to individualise what you do with it … But sometimes using a score or a tool is a way into a service.” (PC4, nurse practitioner in primary care)

It was also recognised by one General Practitioner (GP) that although there is familiarity with risk assessment in primary care, there needs to be caution that the system is not overwhelmed with such tools:

For peer review only - http://bmjopen.bmj.com/site/about/guidelines.xhtml

"I do quite like risk profiling. I think we went a little bit crazy with the risk profiling. And there feels to be a lot of competing risk profiling tools, that we're getting a little bit inundated with at the moment … So I think anything like this, I love, if it can be incorporated and brought on to an individual and needs level - so you can think about caring, identifying risk and needs for an individual - would feel great for me" (PC2, General Practitioner)

Secondary care provides specialist input

Stroke care clinicians discussed the facilitators of risk assessment within a specialist setting. This was based on the fact that they felt a responsibility to ensure that post-stroke sequelae are followed up in their specialist services due to the multidisciplinary element of their standard practice and easier access to services. This was particularly important to ensure information could also be given to patients at a time when they may need it the most:

"I think the six-month review tends to be a period of time when the patient's acute side, acute phase of their care has kind of been established, and this is probably the time when they start to recognise problems. And I think it should be within a stroke MDT (multidisciplinary team), not so much focused on by GP's, as such." (SC2, Stroke specialist nurse)

"Well, you need the right support. You need people that actually understand stroke. So I think it would have to be delivered by stroke healthcare professionals. And I think you get so much information when you're initially an inpatient, I think maybe that's not the best place to do it … Yeah, it's a big thing to be told that you might develop dementia in a few years' time, so you need psychologists kind of available

For peer review only - http://bmjopen.bmj.com/site/about/guidelines.xhtml

for if someone needs counselling as a result of that finding. I think it's tricky." (SC6, Stroke physiotherapist)

Collaborative Care

Primary care clinicians commented that there may be a place for both primary and secondary care to work together in identifying those at risk.

"I think primary care would be a completely reasonable place to do that. I guess it's a conversation that could start at diagnosis, at discharge from hospital, like actually, we know that people who have had a stroke are at higher risk of having dementia, these are the things to be aware of, and you know to start that discussion" (PC8, General Practitioner)

Primary and secondary care clinicians felt that such a shared care pathway needed to be formalized to reduce the risk of individuals falling into gaps in care:

"… even if it was picked up in secondary care it's still going to be primary care where most of the management is occurring. So I think it being identified at the six-month follow-up, but then there being a formal sort of mechanism, in which primary care pick it up and process it, would be fine. (PC3, General Practitioner)

"I don't mind where work is done, provided that it is done in a structured and standardised way. If that be, if that can be in primary care that is really good, because that is the long-term follow-up, long-term support, integrating the community … just as long as it can be delivered in a systematic way, and people don't fall through gaps or get inconsistent care." (SC3, Stroke consultant)

For peer review only

Further, the process of communication between primary and secondary care could also be used in the diagnostic process. It was felt that repeated assessments could help facilitate diagnosis by identifying trends in symptoms:

"You can measure a trend, can't you, if you're using something and measuring something, you can look at a trend. So if its, depends on the type of tool, I guess. But if you did it at you know at the six months review date and then we did it subsequently a year later in primary care, you would see any changes or decline or improvement. So it's a way of, it's a way of monitoring a trend on how they're doing, I guess. So I don't, I don't see any reason why it couldn't be done in both and used across both. I don't think we use enough across both." (PC4, nurse practitioner in primary care)

Clinician Views: Barriers to Risk Assessment Focusses on the Outcome

<u>*Limited Interventions Available*</u>

Similar to the perspectives of carers, clinicians recognised the anxiety that a risk assessment process might generate and felt that it should be a personal choice to undertake an assessment because of the perceived lack of intervention:

"Yeah, I think I would, I would have degree of anxiety, especially given that the measures that we're putting in place are … that we could put in place are largely supportive rather than preventative … I would be less confident that I could be giving my patient advice to say, "Well, if we do this, and we do this, and if we do this and you do that then that might move you into an even smaller risk group." (PC3, General Practitioner)

"Outside research trials, I'm not convinced that there is a definitive value in doing that yet. You know if we get really overwhelming evidence that it's amenable to

For peer review only

intervention so you know there's all the theory about blood pressure, and statins, and all the rest of that, but my reading of the evidence on all of that at the moment is that the jury is out whether it makes a difference to cognitive function. So yeah, I'm not convinced that identifying risk, unless you've got a something you can do about it, is actually sensible." (SC4, Stroke consultant)

Anxiety around a potential diagnosis of dementia

In recognising the anxiety that this process may generate, one clinician also commented on the fact that patients may not be willing to engage in conversation over the subject of dementia and care should be taken when discussing a potential diagnosis of dementia.

"I think it's good if we tell them that we're looking through and saying, "Look, you know there could be a problem here." But for every single patient, again, because it's quite a still a – not a taboo subject – but it's still not something that people want to talk about … I don't know whether it would be used on every single 'per', you know what I mean, like, everybody." (SC5, Stroke specialist nurse)

No Change from Standard Practice

The majority of clinical participants wanted to know, not only what the outcome of the risk assessment would be, but also the resulting care the patient would receive. As part of current routine clinical care, all stroke survivors are offered annual reviews in order to ensure their vascular risk factors e.g. blood pressure and cholesterol are well controlled. In terms of reducing risk, one primary care physician expressed concerns as to what the benefit would to the individual if risk factor modification was already in place anyway particularly with regards to the emotive side of a potential dementia diagnosis. A secondary care specialist questioned the value when there

For peer review only

was seemingly limited interventions that could be implemented besides managing their cardiovascular risk:

"I guess you've got to be very clear about what it is that you're going to be doing differently for them. So I can see the value if you use a tool for kind of primary prevention, then you're kind of selecting a group of patients out to do something particular with, but I just wonder what would be different about what you do with a risk assessment tool for people who have already had a stroke, when really you know already that it is all about managing their cardiovascular risk so I'm not sure that you would be doing anything different for them." (PC8, General Practitioner)

"Many people will not know of the association between dementia and stroke and many people would not want to know if they were at risk of dementia and again, if you're identifying somebody at risk of a condition that you can't do anything about, what's the right stage to, to do that? However, many of the things you need to do in terms of people being at risk of dementia are the same of the general cardiovascular. So, I'm not sure that there is anything additional that needs to be done about reducing people's risk for dementia over and above general cardiovascular risk." (SC3, Stroke consultant)

Discussion

Main Findings

This is the first study to explore key stakeholders' - stroke survivors, family carers and primary and secondary care clinicians - views on the use of a risk assessment process to predict future dementia in stroke-survivors. It is clear that some of the participants interviewed believed that risk assessment could be of clinical use but raised concerns about it being mandatory. Clinicians highlighted both

For peer review only

the benefits of collaborative and individual (i.e. primary or secondary) care if dementia risk assessment for stroke survivors was to be implemented.

Clinician facilitators suggest benefits in either primary or secondary care settings but also in a collaborative model of care between the two. This latter finding echoes recommendations from the UK Intercollegiate Stroke Working Party for a collaborative care model, linking community and specialist care, with the aim of integrated long-term follow-up for those presenting neuropsychological problems[16]. Although both primary and secondary care clinicians could see the benefits of carrying this assessment in their own specialties, patients and carers in this study valued their relationship with their GP. Further, primary care clinicians themselves are familiar with the process of risk assessment. A recent survey of primary care physicians found that they were also keen to implement a dementia risk assessment strategy to assist in earlier identification[21]. However, potential barriers have been identified in previous studies such as system-related factors (lack of support, time constraints)[22 23] and training in dementia[23], would need to be addressed. Risk assessment is an objective process requiring specific individual variables e.g. age, gender, education. Such data is readily available in primary care in many countries where electronic medical record systems are in place. Further, GPs are already asked to assess cardiovascular risk as part of routine clinical care [24]. However, some GPs themselves do not like using risk assessment tools particularly as the tools do not provide the support needed in communication[25]. Training in communicating the risk assessment process particularly in the context of dementia would be required if this were to be implemented in clinical practice.

Clinician participants were concerned about whether risk assessment would actually change standard practice. In a stroke population, it is unclear whether

For peer review only - http://bmjopen.bmj.com/site/about/guidelines.xhtml

identifying those at risk would achieve any additional benefit from a risk factor modification point of view. This is because stroke-survivors already receive annual community follow-up with particular focus on vascular risk factor modification. However, current evidence suggests that development of post-stroke dementia is more than just about vascular risk and would require a different approach e.g. psychological support, cognitive preservation strategies and additional resources. Results from several trials, assessing whether vascular-based interventions can reduce dementia risk, have been largely disappointing[26 27]. These results suggest that perhaps an individual's risk of post-stroke cognitive impairment and dementia includes risk factors beyond vascular risk and/or that the disease has a different mechanism such as inflammatory changes in the cerebral vasculature triggered by stroke or related to small vessel disease.

Currently population screening for dementia is not recommended due to a lack of evidence evaluating risks and benefits[28], despite positive views from older adults[29]. Risk assessment can target high-risk groups rather than the general population. Recent evidence has found a decline in age-specific incidence of dementia, particularly in high-income countries, suggesting that rising levels of education and modifying cardiovascular risk may have driven a decline in dementia risk[30 31]. Indeed, the importance of modifiable risk factor reduction for dementia was reported in the World Alzheimer Report (2014)[32] and around a third of Alzheimer's disease cases worldwide might be attributable to modifiable risk factors[33]. Risk assessment tools utilize these modifiable risk factors to predict risk. Similar to other branches of medicine where risk assessment is utilized to predict risk of a future illness, it would be hoped that this approach could reduce one's risk of future dementia. Stroke affects more than 100,000 people in the UK per year[34], creating a

For peer review only

For peer review only - http://bmjopen.bmj.com/site/about/guidelines.xhtml

large population with cognitive deficits and/or at high risk of future decline who may benefit from risk assessment for dementia. However, participant groups in this study, particularly clinicians, reported that given the potential ramifications of risk assessment, individuals should be given the choice of whether to undergo assessment. Stroke-survivors were generally positive about such an approach but agreed that it should be up to the individual and the family rather than applied universally. Participants in this study recognised the anxiety this process could generate particularly when the perceived possible interventions for dementia are limited. The National Institute for Health and Care Excellence have recently updated their guidance and have concluded that case finding should only be conducted as part of a clinical trial, which also provides an intervention[35]. Therefore, careful discussion needs to be adopted with the patient and their carers before undertaking such a process in any setting.

Clinical Implications

Case finding for dementia involves actively assessing individuals at risk of a future dementia illness, which at present is only recommended in clinical trial settings due to a lack of post-assessment intervention[36]. Once a suitable intervention is found however, the views of those conducting the assessment and the recipients of such an assessment will need to be assessed. From this study we have identified the priorities according to each stakeholder group which would need to be addressed prior to clinical implementation in the future.

Limitations

The participants in this study came from one area of England and were Caucasian. Patient participants were also well enough to attend outpatient assessment clinics.

For peer review only

Future studies could look to explore views in other populations including views from minority ethnic groups, patients with more severe stroke-related impairments and different service models. Due to familiarity, it is recognized that clinicians expanded more around the risk assessment process. Despite this being the case, patients and carers were given the opportunity to understand the concept of risk assessment as part of the interview process but the emphasis on a need for a diagnosis and good care was what was important for them. Participants were also aware that the interviewer was also a primary care clinician, which may have the potential to introduce bias into participant responses. This is because a clinician interviewer may be viewed as an expert and judge in clinical decision making and moral judgements made[37]. On the other hand interviews tend to be broader in scope and richer in data when conducted by a clinician researcher [37]. Further, both clinical and non-clinical members contributed to the analysis of the data to minimize the effect this may have had.

Conclusions and Future Research

Timely recognition of those at risk of dementia is crucial to enable individuals early access treatment and support. Although dementia screening after stroke is not yet advocated on preventative grounds, assessing risk has some potential benefits for individuals who make an informed choice to participate. There would need to be better cohesiveness of communication between primary and secondary care, with more support placed in the community. Further, it should be recognised that if risk assessment were to be incorporated into clinical practice, this will potentially place additional burdens on a dementia diagnostic service which is already overstretched. Next steps are to identify which tool to use, how best to manage those who are deemed high-risk individuals and whether there are any interventions, which can

For peer review only - http://bmjopen.bmj.com/site/about/guidelines.xhtml

reduce their risk. Future studies will need to look specifically at what factors put a stroke-survivor at risk that could be potentially modified and also whether there are specific interventions suitable to a post-stroke population to reduce risk.

Acknowledgements:

The authors would like to thank the participant advisory group for their advice on the study materials used.

List of Abbreviations

**GP**	General Practitioner
**UK**	United Kingdom

Declarations

Author Contributions:

ET conceived the framework for this study. ET collected, analysed and interpreted the data. ET prepared the manuscript for submission.

CE helped to conceive the framework for this study and assisted with the analysis of the data and contributed to the drafting of the manuscript. CE also critically reviewed and edited the manuscript.

CP helped to conceive the framework for this study, assisted with the analysis of the data and critically reviewed and edited the manuscript.

BS helped to conceive the framework for this study, assisted with the analysis of the data and critically reviewed and edited the manuscript.

LR helped to conceive the framework for this study, assisted with the analysis of the data and critically reviewed and edited the manuscript.

For peer review only - http://bmjopen.bmj.com/site/about/guidelines.xhtml

Funding:

Eugene Tang is supported by a NIHR Doctoral Research Fellowship (DRF-2015-08-006).

Louise Robinson is supported by a National Institute for Health Research professorship (NIHR-RP-011-043) and a NIHR Senior Investigator award (NF-SI-0616-10054).

Consent for Publication:

All participants in the study have provided informed written consent. No identifiable personal information has been used.

Competing Interests:

LR reports grants from NIHR Professorship award, grants from NIHR Senior Investigator award, outside the submitted work. The remaining authors declare that they have no competing interests.

Disclaimer:

This paper presents independent research funded by the National Institute for Health Research (NIHR). The views expressed are those of the author(s) and not necessarily those of the NHS, the NIHR or the Department of Health.

Data Sharing Statement:

No further data will be made available.

For peer review only

References

1. Prince M, Wimo W, Guerchet M, et al. World Alzheimer Report 2015: The Global Impact of Dementia, An Analysis of Prevalence, Incidence, Cost and Trends. 2015.
2. Savva GM, Stephan BC, Alzheimer's Society Vascular Dementia Systematic Review G. Epidemiological studies of the effect of stroke on incident dementia: a systematic review. *Stroke; a journal of cerebral circulation* 2010;41(1):e41-6. doi: http://dx.doi.org/10.1161/STROKEAHA.109.559880
3. Pendlebury ST, Rothwell PM. Prevalence, incidence, and factors associated with pre-stroke and post-stroke dementia: a systematic review and meta-analysis. *The Lancet Neurology* 2009;8(11):1006 - 18.
4. Douiri A, Rudd AG, Wolfe CD. Prevalence of poststroke cognitive impairment: South London Stroke Register 1995-2010. *Stroke; a journal of cerebral circulation* 2013;44(1):138-45. doi: 10.1161/strokeaha.112.670844 [published Online First: 2012/11/15]
5. Feigin VL, Forouzanfar MH, Krishnamurthi R, et al. Global and regional burden of stroke during 1990-2010: findings from the Global Burden of Disease Study 2010. *Lancet* 2014;383(9913):245-54. [published Online First: 2014/01/23]
6. Prince M, Ali GC, Guerchet M, et al. Recent global trends in the prevalence and incidence of dementia, and survival with dementia. *Alzheimer's research & therapy* 2016;8(1):23. doi: 10.1186/s13195-016-0188-8 [published Online First: 2016/07/31]
7. Stephan BCM, Kurth T, Matthews FE, et al. Dementia risk prediction in the population: are screening models accurate? *Nat Rev Neurol* 2010;6(6):318 - 26.
8. Tang EY, Harrison SL, Errington L, et al. Current Developments in Dementia Risk Prediction Modelling: An Updated Systematic Review. *PloS one* 2015;10(9):e0136181. doi: 10.1371/journal.pone.0136181 [published Online First: 2015/09/04]
9. Kandiah N, Chander RJ, Lin X, et al. Cognitive Impairment after Mild Stroke: Development and Validation of the SIGNAL2 Risk Score. *Journal of Alzheimer's disease : JAD* 2016;49(4):1169-77. doi: 10.3233/jad-150736 [published Online First: 2015/11/26]
10. Lin JH, Lin RT, Tai CT, et al. Prediction of poststroke dementia. *Neurology* 2003;61(3):343-8. [published Online First: 2003/08/13]
11. Stephan BC, Minett T, Muniz Terrera G, et al. Dementia prediction for people with stroke in populations: is mild cognitive impairment a useful concept? *Age and ageing* 2014 doi: 10.1093/ageing/afu085 [published Online First: 2014/07/09]
12. Chander RJ, Lam BYK, Lin X, et al. Development and validation of a risk score (CHANGE) for cognitive impairment after ischemic stroke. *Scientific reports* 2017;7(1):12441. doi: 10.1038/s41598-017-12755-z [published Online First: 2017/10/01]
13. Siontis GC, Tzoulaki I, Siontis KC, et al. Comparisons of established risk prediction models for cardiovascular disease: systematic review. *BMJ (Clinical research ed)* 2012;344:e3318.
14. Johnston SC, Rothwell PM, Nguyen-Huynh MN, et al. Validation and refinement of scores to predict very early stroke risk after transient ischaemic attack. *Lancet* 2007;369(9558):283-92. doi: 10.1016/s0140-6736(07)60150-0 [published Online First: 2007/01/30]
15. Tang EYH, Price C, Stephan BCM, et al. Gaps in care for patients with memory deficits after stroke: views of healthcare providers. *BMC health services research* 2017;17(1):634. doi: 10.1186/s12913-017-2569-5 [published Online First: 2017/09/10]
16. Intercollegiate Stroke Working Party. National Clinical Guideline for Stroke. 2016 [published Online First: Fifth Edition]
17. Braun V, Clarke V. Using thematic analysis in psychology. *Qualitative Research in Psychology* 2006;3(2):77-101. doi: 10.1191/1478088706qp063oa
18. Glaser B. The constant comparison method of qualitative analysis. *Soc Probl* 1965;12:436-45.

For peer review only - http://bmjopen.bmj.com/site/about/guidelines.xhtml

19. Ritchie J, Spencer L, O'Connor W. Carrying out qualitative analysis In: Ritchie J, Lewis J, editors. Qualitative Research Practice: A guide for social science students and researchers. London: SAGE 2003:219 - 262.
20. O'Brien BC, Harris IB, Beckman TJ, et al. Standards for reporting qualitative research: a synthesis of recommendations. *Academic medicine : journal of the Association of American Medical Colleges* 2014;89(9):1245-51. doi: 10.1097/acm.0000000000000388 [published Online First: 2014/07/01]
21. Tang EY, Birdi R, Robinson L. Attitudes to diagnosis and management in dementia care: views of future general practitioners. *International psychogeriatrics* 2016:1-6. doi: 10.1017/s1041610216001204 [published Online First: 2016/08/10]
22. Koch T, Iliffe S. Rapid appraisal of barriers to the diagnosis and management of patients with dementia in primary care: a systematic review. *BMC Fam Pract* 2010;11:52. doi: 10.1186/1471-2296-11-52 [published Online First: 2010/07/03]
23. Chithiramohan A, Iliffe S, Khattak I. Identifying barriers to diagnosing dementia following incentivisation and policy pressures: General practitioners' perspectives. *Dementia (London, England)* 2016 doi: 10.1177/1471301216682625 [published Online First: 2016/12/13]
24. Hippisley-Cox J, Coupland C, Brindle P. Development and validation of QRISK3 risk prediction algorithms to estimate future risk of cardiovascular disease: prospective cohort study. *BMJ (Clinical research ed)* 2017;357:j2099. doi: 10.1136/bmj.j2099 [published Online First: 2017/05/26]
25. Barfoed BL, Jarbol DE, Paulsen MS, et al. GPs' Perceptions of Cardiovascular Risk and Views on Patient Compliance: A Qualitative Interview Study. *International journal of family medicine* 2015;2015:214146. doi: 10.1155/2015/214146 [published Online First: 2015/10/27]
26. Matz K, Teuschl Y, Firlinger B, et al. Multidomain Lifestyle Interventions for the Prevention of Cognitive Decline After Ischemic Stroke: Randomized Trial. *Stroke; a journal of cerebral circulation* 2015;46(10):2874-80. doi: 10.1161/strokeaha.115.009992 [published Online First: 2015/09/17]
27. Bath PM, Scutt P, Blackburn DJ, et al. Intensive versus Guideline Blood Pressure and Lipid Lowering in Patients with Previous Stroke: Main Results from the Pilot 'Prevention of Decline in Cognition after Stroke Trial' (PODCAST) Randomised Controlled Trial. *PloS one* 2017;12(1):e0164608. doi: 10.1371/journal.pone.0164608 [published Online First: 2017/01/18]
28. Moyer VA. Screening for cognitive impairment in older adults: U.S. Preventive Services Task Force recommendation statement. *Annals of internal medicine* 2014;160(11):791-7. doi: 10.7326/m14-0496 [published Online First: 2014/03/26]
29. Justiss MD, Boustani M, Fox C, et al. Patients' attitudes of dementia screening across the Atlantic. *International journal of geriatric psychiatry* 2009;24(6):632-7. doi: 10.1002/gps.2173 [published Online First: 2008/12/31]
30. Langa KM. Is the risk of Alzheimer's disease and dementia declining? *Alzheimer's research & therapy* 2015;7(1):34. doi: 10.1186/s13195-015-0118-1 [published Online First: 2015/03/31]
31. Larson EB, Yaffe K, Langa KM. New insights into the dementia epidemic. *The New England journal of medicine* 2013;369(24):2275-7. doi: 10.1056/NEJMp1311405 [published Online First: 2013/11/29]
32. Prince M, Albanese E, Guerchet M, et al. Dementia and Risk Reduction: An Analysis of Protective and Modifiable Factors. 2014.
33. Norton S, Matthews FE, Barnes DE, et al. Potential for primary prevention of Alzheimer's disease: an analysis of population-based data. *The Lancet Neurology* 2014;13(8):788-94. doi: 10.1016/s1474-4422(14)70136-x [published Online First: 2014/07/18]
34. Stroke Association. State of the nation: stroke statistics. 2017. https://www.stroke.org.uk/resources/state-nation-stroke-statistics (accessed 14th August 2017).

For peer review only - http://bmjopen.bmj.com/site/about/guidelines.xhtml

35. Faircloth CA, Boylstein C, Rittman M, et al. Sudden illness and biographical flow in narratives of stroke recovery. *Sociology of health & illness* 2004;26(2):242-61. doi: 10.1111/j.1467-9566.2004.00388.x [published Online First: 2004/03/19]
36. National Institute for Health and Care Excellence. Dementia: assessment, management and support for people living with dementia and their carers 2018. https://www.nice.org.uk/guidance/ng97.
37. Chew-Graham CA, May CR, Perry MS. Qualitative research and the problem of judgement: lessons from interviewing fellow professionals. *Fam Pract* 2002;19(3):285-9. [published Online First: 2002/04/30]

For peer review only

Table 1. Interview Participants (Patients and Carers)

Unique Identifier (Patients and Carers)	Role	Gender	Age	Follow-up Interview Conducted
P1	Stroke-survivor	Female	80	No
P2	Stroke-survivor	Female	76	Yes
P3	Stroke-survivor	Female	72	Yes
P4	Stroke-survivor	Male	75	Yes
P5	Stroke-survivor	Male	80	Yes
P6	Stroke-survivor	Male	74	Yes
P7	Stroke-survivor	Female	73	Yes
P8	Stroke-survivor	Female	82	Yes
P9	Stroke-survivor	Male	84	No
P10	Stroke-survivor	Male	79	Yes
C1	Carer of P1 (Husband)	Male	79	No
C2	Carer of P4 (Wife)	Female	79	Yes
C3	Carer of P5 (Daughter)	Female	57	Yes
C4	Carer of P6 (Wife)	Female	71	Yes
C5	Carer of P8	Female	60	

For peer review only

	(Daughter)			

For peer review only

Table 2. Interview Participants (Clinicians)

**Unique Identifier (Clinicians)**	**Role**	**Gender**
SC1	Stroke Consultant	Female
SC2	Stroke Specialist Nurse	Female
SC3	Stroke Consultant	Female
SC4	Stroke Consultant	Male
SC5	Stroke Specialist Nurse	Female
SC6	Stroke Physiotherapist (Rehabilitation)	Female
SC7	Stroke Physiotherapist (Acute Care)	Female
SC8	Stroke Occupational Therapist (Acute Care)	Male
SC9	Stroke Occupational Therapist (Rehabilitation)	Female
PC1	General Practitioner with Specialist Interest in Dementia	Male
PC2	General Practitioner	Male
PC3	General Practitioner	Female
PC4	Nurse Practitioner in primary care	Female
PC5	General Practitioner	Female
PC6	Practice Nurse	Female

For peer review only

PC7	Nurse Practitioner in primary care	Female
PC8	General Practitioner	Female

For peer review only

Supplementary Table 1

Standards for Reporting Qualitative Research Checklist[1]

No.	Topic	Item	Page(s)
	**Title and abstract**		
S1	Title	Concise description of the nature and topic of the study Identifying the study as qualitative or indicating the approach (e.g., ethnography, grounded theory) or data collection methods (e.g., interview, focus group) is recommended	1
S2	Abstract	Summary of key elements of the study using the abstract format of the intended publication; typically includes background, purpose, methods, results, and conclusions	2 - 3
	**Introduction**		
S3	Problem formulation	Description and significance of the problem/phenomenon studied; review of relevant theory and empirical work; problem statement	5 – 6
S4	Purpose or research question	Purpose of the study and specific objectives or questions	6
	**Methods**		
S5	Qualitative approach and research paradigm	Qualitative approach (e.g., ethnography, grounded theory, case study, phenomenology, narrative research) and guiding theory if appropriate; identifying the research paradigm (e.g., postpositivist, constructivist/ interpretivist) is also recommended; rationale[b]	8
S6	Researcher characteristics and reflexivity	Researchers' characteristics that may influence the research, including personal attributes, qualifications/experience, relationship with participants, assumptions, and/or presuppositions; potential or actual interaction between researchers' characteristics and the research questions, approach, methods, results, and/or transferability	N/A
S7	Context	Setting/site and salient contextual factors; rationale[b]	6 – 8
S8	Sampling strategy	How and why research participants, documents, or events were selected; criteria for deciding when no further sampling was necessary (e.g., sampling saturation); rationale[b]	7 - 8
S9	Ethical issues pertaining to human subjects	Documentation of approval by an appropriate ethics review board and participant consent, or explanation for lack thereof; other confidentiality and data security issues	6
S10	Data collection methods	Types of data collected; details of data collection procedures including (as appropriate) start and stop dates of data collection and analysis, iterative process, triangulation of sources/methods, and modification of procedures in response to evolving study findings; rationale[b]	6 – 7
S11	Data collection instruments and technologies	Description of instruments (e.g., interview guides, questionnaires) and devices (e.g., audio recorders) used for data collection; if/how the instrument(s) changed over the course of the study	7
S12	Units of study	Number and relevant characteristics of participants, documents, or events included in the study; level of participation (could be reported in results)	29 – 32
S13	Data processing	Methods for processing data prior to and during analysis, including transcription, data entry, data management and security, verification of data integrity, data coding, and anonymization/deidentification of excerpts	8 – 9
S14	Data analysis	Process by which inferences, themes, etc., were identified and developed, including the researchers involved in data analysis; usually references a specific paradigm or approach; rationale [b]	8 – 9
S15	Techniques to enhance trustworthiness	Techniques to enhance trustworthiness and credibility of data analysis (e.g., member checking, audit trail, triangulation); rationale[b]	8 – 9
	**Results/findings**		
S16	Synthesis and interpretation	Main findings (e.g., interpretations, inferences, and themes); might include development of a theory or model, or integration with prior research or theory	9 – 19
S17	Links to empirical data	Evidence (e.g., quotes, field notes, text excerpts, photographs) to substantiate analytic findings	9 – 19
	**Discussion**		
S18	Integration with prior work, implications, transferability, and contribution(s) to the field	Short summary of main findings; explanation of how findings and conclusions connect to, support, elaborate on, or challenge conclusions of earlier scholarship; discussion of scope of application/ generalizability; identification of unique contribution(s) to scholarship in a discipline or field	19 – 22
S19	Limitations	Trustworthiness and limitations of findings	22 – 23
	**Other**		
S20	Conflicts of interest	Potential sources of influence or perceived influence on study conduct and conclusions; how these were managed	25
S21	Funding	Sources of funding and other support; role of funders in data collection, interpretation, and reporting	25

Reference

1. O'Brien BC, Harris IB, Beckman TJ, et al. Standards for reporting qualitative research: a synthesis of recommendations. *Academic medicine : journal of the Association of American Medical Colleges* 2014;89(9):1245-51. doi: 10.1097/acm.0000000000000388 [published Online First: 2014/07/01]

The Views of Public and Clinician Stakeholders on Risk Assessment Tools for Post-Stroke Dementia: A Qualitative Study

Journal:	*BMJ Open*
Manuscript ID	bmjopen-2018-025586.R1
Article Type:	Research
Date Submitted by the Author:	08-Nov-2018

Complete List of Authors:	Tang, Eugene; Newcastle University, Institute of Health and Society<br>Exley, Catherine; Northumbria University, Faculty of Health and Life Sciences<br>Price, Christopher; Newcastle University, Institute of Neuroscience, Stroke Research Group<br>Stephan, Blossom; Newcastle University, Institute of Health and Society<br>Robinson, Louise; Newcastle University, Institute of Health and Society

**Primary Subject Heading**:	Qualitative research
Secondary Subject Heading:	Mental health, Neurology, General practice / Family practice
Keywords:	PRIMARY CARE, STROKE MEDICINE, QUALITATIVE RESEARCH, Dementia < NEUROLOGY

SCHOLARONE™ Manuscripts

The Views of Public and Clinician Stakeholders on Risk Assessment Tools for Post-Stroke Dementia: A Qualitative Study

Dr. Eugene Yee Hing Tang[1,2] e.y.h.tang@newcastle.ac.uk

Prof. Catherine Exley[3] catherine.exley@northumbria.ac.uk

Dr. Christopher Price[4] c.i.m.price@newcastle.ac.uk

Dr. Blossom CM Stephan[1,2] blossom.stephan@newcastle.ac.uk

Prof. Louise Robinson[1,2] a.l.robinson@newcastle.ac.uk

[1] Institute of Health and Society, Newcastle University, Baddiley-Clark, Richardson Road, Newcastle upon Tyne, UK, NE2 4AX

[2] Newcastle University Institute of Ageing, Newcastle University, Campus for Ageing and Vitality, Newcastle upon Tyne, UK, NE4 5PL

[3] Faculty of Health & Life Sciences, Northumberland Building, Northumbria University, Newcastle upon Tyne, NE1 8ST

[4] Institute of Neuroscience, Stroke Research Group, Newcastle University

Corresponding author: Dr. Eugene Yee Hing Tang, Institute of Health & Society, Newcastle University, Level 2, Newcastle Biomedical Research Building, Campus for Ageing and Vitality, Newcastle upon Tyne, NE4 5PL, T: 0191 208 8758,

E: e.y.h.tang@newcastle.ac.uk

For peer review only

Abstract

OBJECTIVE: Stroke-survivors are at increased risk of future dementia. Assessment to identify those at high risk of developing a disease using predictive scores has been utilised in different areas of medicine. A number of risk assessment scores for dementia have been developed but none has been recommended for use clinically. The aim of this qualitative study was to assess the acceptability and feasibility of using a risk assessment tool to predict post-stroke dementia.

DESIGN: Qualitative semi-structured interviews were conducted and analysed thematically. Patients and carers were offered interviews at around 6 (baseline) and 12 (follow-up) months post-stroke; Clinicians were interviewed once.

SETTING: The study was conducted in the North-East of England with stroke patients, family carers and healthcare professionals in primary and secondary care.

PARTICIPANTS: Thirty-nine interviews were conducted (17 clinicians and 15 stroke patients and their carers at baseline. Twelve stroke patients and their carers were interviewed at follow-up).

RESULTS: Barriers and facilitators to risk assessment were discussed. For patients and carers the focus for facilitators were based on the outcomes of risk assessment i.e. an assessment could assist with preparation, diagnosis and for reassurance. For clinicians, facilitators were focused on the process i.e. familiarity in primary care, resource availability in secondary care and collaborative care. For barriers, both groups focussed on the outcome including e.g. the anxiety generated from a potential diagnosis of dementia. For patients and carers a further barrier included concerns about how it may affect their recovery. For clinicians there were concerns about limited interventions and how it would be different from standard care.

For peer review only

CONCLUSIONS: Risk assessment for dementia post-stroke presents challenges given the ramifications of a potential diagnosis of dementia. Attention needs to be given to how information is communicated, and strategies developed to support patients and carers if risk assessment has taken place.

For peer review only

Strengths and Limitations of the Study:

- To the best of our knowledge this is the first qualitative study to examine critically the views of stroke patients and their family carers and clinicians about the acceptability and feasibility of a risk assessment approach to assist in earlier identification of post-stroke dementia.
- Understanding stakeholder views on risk assessment for dementia can help inform future strategies if risk assessment for dementia is used to assist with earlier diagnosis.
- Patient participants came from one area of England who were able to attend hospital outpatient departments and so may not represent the views and experiences of those with more severe post-stroke sequelae.
- Clinician participants came from one area of England and so may not represent the views of other service models in other regions of the UK.
- It is recognized that clinicians tended to be more familiar with the process of risk assessment and could elaborate further on the process involved.

For peer review only

Introduction

There is currently no cure for dementia and it is estimated that the worldwide economic burden will rise to US$2 trillion by 2030[1]. It has been suggested that the most powerful way to affect costs is by reducing the numbers of people who develop the illness. This may be facilitated by prediction of individual risk for the disease. Stroke is associated with an increased risk of dementia and cognitive impairment[2-4]. A recent meta-analysis found that both prevalent and incident strokes are strong independent risk factors for dementia[5]. Stroke incidence and numbers of stroke-survivors are likely to increase due to simultaneous ageing populations and declining stroke mortality rates[6]. Given that the incidence of dementia increases exponentially with age[1 7], this will mean that post-stroke dementia will also become increasingly prevalent. It will therefore be important to identify those at greatest risk of developing dementia following stroke in order to implement strategies to reduce risk. In general, strategies to reduce risk of dementia may include management of cardiovascular risk factors e.g. smoking, diabetes as well as regular physical activity[8].

Risk prediction models for dementia to identify those at higher risk have been developed in whole populations[9 10] with some models specifically developed to predict cognitive impairment and dementia in stroke populations[11-14]. These stroke-specific models predict dementia or cognitive impairment over a relatively short time period (up to 18 months[14]). In spite of the expanding research in this field, none of the dementia risk prediction tools have been clinically implemented. Further, no studies have assessed the feasibility or acceptability of implementing such a strategy in a stroke population. Although risk models are currently used in everyday clinical practice in other branches of medicine, in particular prevention of cardiovascular[15] and cerebrovascular[16] disease, it is unclear how clinicians would feel about using a

For peer review only

similar strategy to predict dementia, particularly given the stigma surrounding the diagnosis and perceived limited interventions and increased awareness of cognitive difficulties that patients and carers may have following stroke. Further, no studies have evaluated whether using risk assessment tools for dementia would be acceptable to stroke patients themselves.

This paper presents findings from a qualitative study conducted with patients, carers and clinicians, which sought to critically examine their views about the acceptability and feasibility of using risk prediction models in post-stroke care to identify those at greatest risk of future dementia.

Methods

Patient and Public Involvement (PPI)

Patients and members of the public have been involved in the development of this study from the beginning of the proposal. A participant advisory group also oversees the work conducted and annual face-to-face meetings are held to inform them of the study findings. The participant advisory group consists of members from a stroke research patient and carer panel, an organisation aimed at capturing public views about research and from a dementia and neurodegeneration specialty PPI group. The same group reviewed the study materials to ensure suitability particularly for stroke-survivors and their family carers.

Ethical Approval

For peer review only

The study was conducted in the North East of England. Ethical Approval was obtained from the London – Hampstead Research Ethics Committee (reference 16/LO/0133). Participants provided informed written consent prior to the interview.

Patient and Carer Sampling

Patients and carers were purposively sampled from stroke clinics i.e. to ensure a mix of genders and a range carers were recruited. As part of routine clinical practice in United Kingdom (UK) stroke services, all stroke-survivors are invited to a specialist review at six months after the event which includes a general enquiry about memory concerns[17]. If the patient reported any subjective memory concerns at the clinic and was over the age of 60, the stroke specialist nurse would provide further study information. Family carers were also recruited if they were involved in the stroke-survivor's care, for example, if they attended the clinic appointment with them. If potential participants were interested in taking part in the study, their details were passed onto the research team. On receipt of this information one researcher (EYHT) would make contact with the patient or carer. He would provide detailed information and an opportunity to ask questions about the study. Following their agreement to participate in the study, participants were asked to take part in an interview immediately following their six-month review and/or around six months later.

Clinician Sampling

General Practitioners (GPs) and secondary care clinicians (stroke consultants and specialist nurses) in the North East of England were contacted to participate in the study. Participants were given an opportunity to ask further questions. Clinicians

For peer review only

were purposively sampled to ensure that a broad range of care professionals in both primary and secondary care were recruited.

Data collection

Interviews were conducted between April 2016 and August 2017 by one researcher (EYHT) who is a medical doctor. The topic guide was initially derived from relevant literature and expert clinical views within the research team. It was designed to be iterative to enable any topics, which had not been previously identified, to be pursued in subsequent interviews. Face to face semi-structured interviews were conducted with all but one participant (clinician) who had a telephone interview. The patient and family carer were interviewed individually or in pairs as requested by participants. Clinicians were interviewed individually. The interviews focussed on the benefits and challenges of improving earlier diagnosis of dementia after stroke. This included specific questions on the delivery of this assessment (e.g. who should carry it out), what variables could be used and how best to manage the outcome. Alongside this, the interviews also sought the views of stakeholders on the care experience of post-stroke individuals with memory problems from clinicians, patients and carers. The interviews also looked to understand the impact of post-stroke memory problems on patients and carers. These views on care experience from clinicians[18], patients and carers[19] have been reported elsewhere. The impact of post-stroke memory problems on patients and carers will be reported separately. This paper reports the views of clinicians, patients and carers on risk assessment only. The process of risk assessment was described to participants. This was further emphasised with examples of published tools in order to highlight examples of variables used to ensure participant understanding of the process. Informed written consent was obtained from all participants prior to the interview commencing. All

For peer review only - http://bmjopen.bmj.com/site/about/guidelines.xhtml

interviews were audio-recorded and then transcribed verbatim. To protect participant anonymity, unique identifiers were used throughout the process with identifiable personal data removed.

Data analysis

Interview data was analysed using thematic analysis[20] following the principles of constant comparative methods[21]. We ceased data collection when the researcher felt that data saturation occurred. This was defined as being when a full understanding of the participant's perspective[22] and also "informational redundancy" had been reached[23]. One researcher (EYHT) familiarised himself with the dataset and subsequently coded the transcripts line-by-line. Initially, a small subset of transcripts were analysed to identify initial themes and these were discussed between CE and EYHT. Data collection and analysis was iterative and as interviews progressed, further analysis led to new themes emerging and refinement of existing themes and subthemes, which were subsequently grouped into broad categories to facilitate interpretation. The wider team (EYHT, CE, LR, BS and CP) discussed and agreed on the final categories which are presented below. For patient and carer interviews, where follow-up interview data was also obtained, these were analysed as separate interviews to assess for any change in views over time. Data analysis continued after fieldwork had ceased. There was particular focus to understand what was important to patients, carers and clinicians. Data analysis was facilitated by a data software handing package (NVivo version 11). The paper conforms to the Standards for Reporting Qualitative Research checklist[24] (please see supplementary table 1).

Results

For peer review only - http://bmjopen.bmj.com/site/about/guidelines.xhtml

In total, 30 baseline (6 month) interviews were conducted, analysed and compared including: 15 patient and carer interviews (see table 1) and 17 primary and secondary care clinician interviews (see table 2). Two pairs of participants were interviewed together at baseline. Eight stroke-survivors and four carers agreed to a further follow-up interview six months later with nine interviews completed. Three pairs of participants were interviewed together at follow-up. One stroke-survivor declined further follow-up, another stroke-survivor and carer were not followed up due to medical reasons. The data from this study suggest that in terms of risk assessment facilitators and barriers exist to implementation. Whereas patient facilitators focussed on the outcome of the risk assessment, clinicians focussed more on the process of risk assessment for facilitators. Both groups discussed some potential barriers associated with risk assessment focussing on the outcome.

Patient and Carer Views: Facilitators to Risk Assessment Focuses on the Outcome of Assessment

When stroke-survivors and carers discussed the concept of risk assessment, the overarching theme was that an assessment outcome was what was important, irrespective of the process and clinicians involved. Participants focussed on several areas of why the outcome was important to them.

For Preparation

Some stroke-survivors were generally positive about receiving a risk assessment for dementia. One stroke-survivor acknowledged that a diagnosis was something that could enable individuals to prepare themselves both at baseline and subsequently at follow-up interview:

For peer review only - http://bmjopen.bmj.com/site/about/guidelines.xhtml

"It's the same as knowing and not knowing, if you know that something is approaching. Not everybody is the same with the problem. You might be able to deal with it in a different way or the person supporting you, the nurse or whoever, might be able to find a different way or a more positive way of managing it." (P6, male, stroke-survivor at follow-up interview)

Similarly, for carers, there was the emphasis on what could be done following the assessment. One carer emphasized the importance of looking after the whole person, and, how earlier recognition of a potential dementia diagnosis could ensure strategies were in place to help the individual:

"But I think, if you look at the whole thing of this care of this person, if we knew earlier that you know the chances are that your memory is going to get bad and you are going to go into dementia or whatever, then we can start thinking, "Right, well let's prop it up, let's think of ways in helping your memory as it is, to maintain the level it is before you've got no choice, it's going to get worse." You know, maintaining what you've got and different ways of maintaining it, I think that would help." (C5, female carer (daughter) of stroke-survivor)

<u>*For Timely Diagnosis*</u>

For some stroke-survivors it did not matter who was performing the risk assessment for dementia or where it was undertaken. What was important was that the diagnosis was reached at the right time:

"I wouldn't say it matters, as long as it's diagnosed at the right time." (P5, male stroke-survivor)

To enable this, when discussing who should perform the risk assessment, carer participants felt that primary care and the community were regarded as being optimal

For peer review only - http://bmjopen.bmj.com/site/about/guidelines.xhtml

because of the existing GP-patient relationship. This is because the GP has an overall view of the individual's care:

"I think if you've got a good relationship with your GP I think it should be that, it should be them. Yeah, because you know you trust them you build up a relationship with them so I think that probably, for me that would be the one." (C4, female carer of stroke-survivor)

For Reassurance

When stroke-survivor participants were asked about a structured risk assessment process, a further participant reported that the outcome could also ensure some reassurance, either that their symptoms were not related to a dementia diagnosis or that a diagnosis of dementia would be accompanied by support information:

"I think it's reassurance a lot of reassurance with people. You have to give them that to tell them, that "We are there with you. We're going to be helping you." And thats you know, I think that's a good thing." (P2, female stroke-survivor)

Patient and Carer Views: Barriers to Risk Assessment Focuses on the Outcome of Assessment

Anxiety around a potential diagnosis of dementia

Some carers commented on how the outcome from risk assessment could generate worry and anxiety because of the potential diagnosis of dementia:

"To be honest, I don't know if it would help somebody saying, "You're like this, you're upset because you're like this now, but we actually think you're going to get much worse." Do you know what I mean?" (C3, female carer (daughter) of stroke-survivor)

For peer review only - http://bmjopen.bmj.com/site/about/guidelines.xhtml

This person's opinion did not change when she was followed-up six months later. The participant's focus was again on worrying about what could develop and how not knowing about one's risk would actually be more preferable:

"If you could find out and then say, "Right, we've got this medication, or something, that can help you," maybe. But if they're just going to tell you, and then you've got this hanging over your head, and you're thinking, "When is it going to start?" and then you'd be thinking you'd forget something and you'd think, "Oh, that's it, it's coming", which it would be quite normal if you hadn't had that diagnosis, you'd think, "Well I just forgot something, everybody does that." (C3, female carer (daughter) of stroke-survivor at follow-up interview)

However, one carer felt that despite the worry a potential diagnosis may generate, the benefit of this would be to find strategies to maintain cognitive function:

"I think if you had earlier diagnosis, then you would be sort of prepared before things got difficult to handle, or before problems arise, that would be a very good thing. The disadvantages as you say, alarming the carers or the patients themselves, "I'm going to lose my mind." Because, particularly in the older generation, that's a big worry to them. It is a big worry, it's a big worry to all of us, but to older people particularly." (C5, female carer (daughter) of stroke-survivor)

Concerns about how it may affect their recovery

Not all stroke-survivors were as keen to engage in risk assessment, as there was emphasis on how this may affect them psychologically particularly when their physical deficits had recovered enough to allow them to return to a more usual routine. Therefore, although diagnosis was felt to be important, whether an individual would like to know was also dependent on their subsequent post-stroke recovery:

For peer review only - http://bmjopen.bmj.com/site/about/guidelines.xhtml

"That's difficult you know because I mean if you have an early diagnosis you know and say, well "It's going to happen" you know but at the moment now I seem to be progressing through, I'm driving now, you know I'm going back to meetings and whatever. I wonder whether an early diagnosis would restrict that." (P4, male stroke-survivor)

This was particularly evident when patients were followed up six-months later. One participant had actually changed her view over time. Although she had initially felt positive about the process, she then changed her mind when questioned on the same process at her follow-up interview:

"I think my thinking has gone the other way for knowing about that. I think it's sad. I think it's a sad thing. I really do, I think it's really sad that for people to know that they're going to be at high risk, it's a sad thing for it to happen to people, and I don't think I'd want to be one of the sad people. I think I'd just want to be, potter along and that's it." (P2, female, stroke-survivor at follow-up interview)

At follow-up interviews participants also felt that risk assessment should be an individual choice because of the ramifications of the assessment outcome i.e. a potential diagnosis of dementia. Although clinicians may deem it to be helpful, the choice to undergo risk assessment needs to be a weighed up, which should negate any calls for it to be made a universally applied process:

"I think, medically speaking, yes. On the other hand, does it give people things to worry about that they wouldn't have worried about if you hadn't done the tests? So, I think it depends really on your personal point of view. Do you want to be, you see I would look on the test as saying, well you're at a low, you've got a low risk so that's great but then if it turned out you'd got a high risk are you going to be more worried

For peer review only - http://bmjopen.bmj.com/site/about/guidelines.xhtml

and less happy than you were before. It's hard to really balance it, isn't it? (P3, female, stroke-survivor at follow-up interview)

Clinician Views: Facilitators to Risk Assessment Focusses on the Process

Clinicians discussed facilitators to risk assessment in terms of how the process may affect the individual and also how the process could be implemented in the future.

When discussing how to implement this process, both primary and secondary care specialists discussed the advantages associated with hosting this process within their own individual teams.

Process familiarity in Primary Care

For primary care, it was about the fact that risk assessment was already a familiar process but that it needed to be individualised:

"I think it's a good tool. We're quite good at using tools, aren't we, but there's always going to be exceptions to the rules and you've got to individualise what you do with it … But sometimes using a score or a tool is a way into a service." (PC4, nurse practitioner in primary care)

It was also recognised by one General Practitioner (GP) that although there is familiarity with risk assessment in primary care, there needs to be caution that the system is not overwhelmed with such tools:

"I do quite like risk profiling. I think we went a little bit crazy with the risk profiling. And there feels to be a lot of competing risk profiling tools, that we're getting a little bit inundated with at the moment … So I think anything like this, I love, if it can be incorporated and brought on to an individual and needs level - so you can think

For peer review only - http://bmjopen.bmj.com/site/about/guidelines.xhtml

about caring, identifying risk and needs for an individual - would feel great for me" (PC2, General Practitioner)

Secondary care provides specialist input

Stroke care clinicians discussed the facilitators of risk assessment within a specialist setting. This was based on the fact that they felt a responsibility to ensure that post-stroke sequelae are followed up in their specialist services due to the multidisciplinary element of their standard practice and easier access to services. This was particularly important to ensure information could also be given to patients at a time when they may need it the most:

"I think the six-month review tends to be a period of time when the patient's acute side, acute phase of their care has kind of been established, and this is probably the time when they start to recognise problems. And I think it should be within a stroke MDT (multidisciplinary team), not so much focused on by GP's, as such." (SC2, Stroke specialist nurse)

"Well, you need the right support. You need people that actually understand stroke. So I think it would have to be delivered by stroke healthcare professionals. And I think you get so much information when you're initially an inpatient, I think maybe that's not the best place to do it … Yeah, it's a big thing to be told that you might develop dementia in a few years' time, so you need psychologists kind of available for if someone needs counselling as a result of that finding. I think it's tricky." (SC6, Stroke physiotherapist)

Collaborative Care

Primary care clinicians commented that there may be a place for both primary and secondary care to work together in identifying those at risk.

For peer review only - http://bmjopen.bmj.com/site/about/guidelines.xhtml

"I think primary care would be a completely reasonable place to do that. I guess it's a conversation that could start at diagnosis, at discharge from hospital, like actually, we know that people who have had a stroke are at higher risk of having dementia, these are the things to be aware of, and you know to start that discussion" (PC8, General Practitioner)

Primary and secondary care clinicians felt that such a shared care pathway needed to be formalized to reduce the risk of individuals falling into gaps in care:

"… even if it was picked up in secondary care it's still going to be primary care where most of the management is occurring. So I think it being identified at the six-month follow-up, but then there being a formal sort of mechanism, in which primary care pick it up and process it, would be fine. (PC3, General Practitioner)

"I don't mind where work is done, provided that it is done in a structured and standardised way. If that be, if that can be in primary care that is really good, because that is the long-term follow-up, long-term support, integrating the community … just as long as it can be delivered in a systematic way, and people don't fall through gaps or get inconsistent care." (SC3, Stroke consultant)

Further, the process of communication between primary and secondary care could also be used in the diagnostic process. It was felt that repeated assessments could help facilitate diagnosis by identifying trends in symptoms:

"You can measure a trend, can't you, if you're using something and measuring something, you can look at a trend. So if its, depends on the type of tool, I guess. But if you did it at you know at the six months review date and then we did it subsequently a year later in primary care, you would see any changes or decline or improvement. So it's a way of, it's a way of monitoring a trend on how they're doing, I

For peer review only - http://bmjopen.bmj.com/site/about/guidelines.xhtml

guess. So I don't, I don't see any reason why it couldn't be done in both and used across both. I don't think we use enough across both." (PC4, nurse practitioner in primary care)

Clinician Views: Barriers to Risk Assessment Focusses on the Outcome

Limited Interventions Available

Similar to the perspectives of carers, clinicians recognised the anxiety that a risk assessment process might generate and felt that it should be a personal choice to undertake an assessment because of the perceived lack of intervention:

"Yeah, I think I would, I would have degree of anxiety, especially given that the measures that we're putting in place are … that we could put in place are largely supportive rather than preventative … I would be less confident that I could be giving my patient advice to say, "Well, if we do this, and we do this, and if we do this and you do that then that might move you into an even smaller risk group." (PC3, General Practitioner)

"Outside research trials, I'm not convinced that there is a definitive value in doing that yet. You know if we get really overwhelming evidence that it's amenable to intervention so you know there's all the theory about blood pressure, and statins, and all the rest of that, but my reading of the evidence on all of that at the moment is that the jury is out whether it makes a difference to cognitive function. So yeah, I'm not convinced that identifying risk, unless you've got a something you can do about it, is actually sensible." (SC4, Stroke consultant)

Anxiety around a potential diagnosis of dementia

For peer review only - http://bmjopen.bmj.com/site/about/guidelines.xhtml

For peer review only

In recognising the anxiety that this process may generate, one clinician also commented on the fact that patients may not be willing to engage in conversation over the subject of dementia and care should be taken when discussing a potential diagnosis of dementia.

"I think it's good if we tell them that we're looking through and saying, "Look, you know there could be a problem here." But for every single patient, again, because it's quite a still a – not a taboo subject – but it's still not something that people want to talk about … I don't know whether it would be used on every single 'per', you know what I mean, like, everybody." (SC5, Stroke specialist nurse)

No Change from Standard Practice

The majority of clinical participants wanted to know, not only what the outcome of the risk assessment would be, but also the resulting care the patient would receive. As part of current routine clinical care, all stroke survivors are offered annual reviews in order to ensure their vascular risk factors e.g. blood pressure and cholesterol are well controlled. In terms of reducing risk, one primary care physician expressed concerns as to what the benefit would to the individual if risk factor modification was already in place anyway particularly with regards to the emotive side of a potential dementia diagnosis. A secondary care specialist questioned the value when there was seemingly limited interventions that could be implemented besides managing their cardiovascular risk:

"I guess you've got to be very clear about what it is that you're going to be doing differently for them. So I can see the value if you use a tool for kind of primary prevention, then you're kind of selecting a group of patients out to do something particular with, but I just wonder what would be different about what you do with a

For peer review only

risk assessment tool for people who have already had a stroke, when really you know already that it is all about managing their cardiovascular risk so I'm not sure that you would be doing anything different for them." (PC8, General Practitioner)

"Many people will not know of the association between dementia and stroke and many people would not want to know if they were at risk of dementia and again, if you're identifying somebody at risk of a condition that you can't do anything about, what's the right stage to, to do that? However, many of the things you need to do in terms of people being at risk of dementia are the same of the general cardiovascular. So, I'm not sure that there is anything additional that needs to be done about reducing people's risk for dementia over and above general cardiovascular risk." (SC3, Stroke consultant)

Discussion

Main Findings

This is the first study to explore key stakeholders' - stroke survivors, family carers and primary and secondary care clinicians - views on the use of a risk assessment process to predict future dementia in stroke-survivors. It is clear that some of the participants interviewed believed that risk assessment could be of clinical use but raised concerns about it being mandatory. Clinicians highlighted both the benefits of collaborative and individual (i.e. primary or secondary) care if dementia risk assessment for stroke survivors was to be implemented.

Clinician facilitators suggest benefits in either primary or secondary care settings but also in a collaborative model of care between the two. This latter finding echoes recommendations from the UK Intercollegiate Stroke Working Party for a collaborative care model, linking community and specialist care, with the aim of

For peer review only - http://bmjopen.bmj.com/site/about/guidelines.xhtml

integrated long-term follow-up for those presenting neuropsychological problems[17]. Although both primary and secondary care clinicians could see the benefits of carrying this assessment in their own specialties, patients and carers in this study valued their relationship with their GP. Further, primary care clinicians themselves are familiar with the process of risk assessment. A recent survey of primary care physicians trainees found that they were also keen to implement a dementia risk assessment strategy to assist in earlier identification[25]. However, potential barriers have been identified in previous studies, such as system-related factors (lack of support, time constraints)[26 27] and training in dementia[27], which would need to be addressed. Risk assessment is an objective process requiring specific individual variables e.g. age, gender, education. Such data is readily available in primary care in many countries where electronic medical record systems are in place. Further, GPs are already asked to assess cardiovascular risk as part of routine clinical care[28]. However, some GPs themselves do not like using risk assessment tools particularly as the tools do not provide the support needed in communication[29]. Training in communicating the risk assessment process, particularly in the context of dementia, would be required if this were to be implemented in clinical practice. Further, some models, particularly those developed in stroke populations[11] may also include variables such as complex imaging data, which will only be available in secondary care and may be difficult to obtain even in specialist settings. If risk assessment were to be conducted in primary care, then the risk assessment models utilising data which can be accessed in primary care, needs to be externally validated in stroke populations to assess their accuracy.

Clinician participants were concerned about whether risk assessment would actually change standard practice. In a stroke population, it is unclear whether

For peer review only - http://bmjopen.bmj.com/site/about/guidelines.xhtml

identifying those at risk would achieve any additional benefit from a risk factor modification point of view. This is because stroke-survivors already receive annual community follow-up with particular focus on vascular risk factor modification. However, current evidence suggests that development of post-stroke dementia is more than just about vascular risk and would require a different approach e.g. psychological support, cognitive preservation strategies and additional resources. Results from several trials, assessing whether vascular-based interventions can reduce dementia risk, have been largely disappointing[30 31]. These results suggest that perhaps an individual's risk of post-stroke cognitive impairment and dementia includes risk factors beyond vascular risk and/or that the disease has a different mechanism such as inflammatory changes in the cerebral vasculature triggered by stroke or related to small vessel disease.

Currently population screening for dementia is not recommended due to a lack of evidence evaluating risks and benefits[32], despite positive views from older adults[33]. Risk assessment can target high-risk groups rather than the general population. Recent evidence has found a decline in age-specific incidence of dementia, particularly in high-income countries, suggesting that rising levels of education and modifying cardiovascular risk may have driven a decline in dementia risk[34 35]. Indeed, the importance of modifiable risk factor reduction for dementia was reported in the World Alzheimer Report (2014)[36] and around a third of Alzheimer's disease cases worldwide might be attributable to modifiable risk factors[37]. Risk assessment tools utilize these modifiable risk factors to predict risk. Similar to other branches of medicine where risk assessment is utilized to predict risk of a future illness, it would be hoped that this approach could reduce one's risk of future dementia. Stroke affects more than 100,000 people in the UK per year[38], creating a

For peer review only - http://bmjopen.bmj.com/site/about/guidelines.xhtml

large population with cognitive deficits and/or at high risk of future decline who may benefit from risk assessment for dementia. However, participant groups in this study, particularly clinicians, reported that given the potential ramifications of risk assessment, individuals should be given the choice of whether to undergo assessment. Stroke-survivors were generally positive about such an approach but agreed that it should be up to the individual and the family rather than applied universally. Participants in this study recognised the anxiety this process could generate particularly when the perceived possible interventions for dementia are limited. The National Institute for Health and Care Excellence have recently updated their guidance and have concluded that case finding should only be conducted as part of a clinical trial, which also provides an intervention[39]. Therefore, careful discussion needs to be adopted with the patient and their carers before undertaking such a process in any setting. In the context of the dementia diagnostic journey, transition from living with an undiagnosed memory problem to being diagnosed with a dementia illness is underpinned by uncertainty[40]. Although risk assessment certainly does not provide any certainty for a dementia illness, the discussions and objective evaluation using the tools may help the individual's process their current condition and assist in the preparation for a potential diagnosis of dementia. Preparation was mentioned by participants in this study as a facilitator for risk assessment.

Clinical Implications

Case finding for dementia involves actively assessing individuals at risk of a future dementia illness, which at present is only recommended in clinical trial settings due to a lack of post-assessment intervention[41]. Once a suitable intervention is found however, the views of those conducting the assessment and the recipients of such an assessment will need to be assessed. Similarly there will be challenges with

For peer review only - http://bmjopen.bmj.com/site/about/guidelines.xhtml

regards to assessment of capacity when performing risk assessment for this at-risk population. It is also important to note that GPs find communicating the diagnosis of dementia difficult[42]. Although risk assessment is not providing a diagnosis of dementia, careful consideration will be required in training health professionals in communicating the concept of risk for a disease such as dementia. From this study we have identified the priorities according to each stakeholder group which would need to be addressed prior to clinical implementation in the future.

Limitations

The participants in this study came from one area of England and were Caucasian. Patient participants were also well enough to attend outpatient assessment clinics. Future studies could look to explore views in other populations including views from minority ethnic groups, patients with more severe stroke-related impairments and different service models. Due to familiarity, it is recognized that clinicians expanded more around the risk assessment process. Despite this being the case, patients and carers were given the opportunity to understand the concept of risk assessment as part of the interview process but the emphasis on a need for a diagnosis and good care was what was important for them. Participants were also aware that the interviewer was also a primary care clinician, which may have the potential to introduce bias into participant responses. This is because a clinician interviewer may be viewed as an expert and judge in clinical decision making and moral judgements made[43]. On the other hand interviews tend to be broader in scope and richer in data when conducted by a clinician researcher [43]. Further, both clinical and non-clinical members contributed to the analysis of the data to minimize the effect this may have had.

For peer review only

Conclusions and Future Research

Timely recognition of those at risk of dementia is crucial to enable individuals early access treatment and support. Although dementia screening after stroke is not yet advocated on preventative grounds, assessing risk has some potential benefits for individuals who make an informed choice to participate. There would need to be better cohesiveness of communication between primary and secondary care, with more support placed in the community. Further, it should be recognised that if risk assessment were to be incorporated into clinical practice, this will potentially place additional burdens on a dementia diagnostic service which is already overstretched. Next steps are to identify which tool to use, how best to manage those who are deemed high-risk individuals and whether there are any interventions, which can reduce their risk. Future studies will need to look specifically at what factors put a stroke-survivor at risk that could be potentially modified and also whether there are specific interventions suitable to a post-stroke population to reduce risk.

Acknowledgements:

The authors would like to thank the participant advisory group for their advice on the study materials used.

List of Abbreviations

**GP**	General Practitioner
**UK**	United Kingdom

Declarations

Author Contributions:

For peer review only

ET conceived the framework for this study. ET collected, analysed and interpreted the data. ET prepared the manuscript for submission.

CE helped to conceive the framework for this study and assisted with the analysis of the data and contributed to the drafting of the manuscript. CE also critically reviewed and edited the manuscript.

CP helped to conceive the framework for this study, assisted with the analysis of the data and critically reviewed and edited the manuscript.

BS helped to conceive the framework for this study, assisted with the analysis of the data and critically reviewed and edited the manuscript.

LR helped to conceive the framework for this study, assisted with the analysis of the data and critically reviewed and edited the manuscript.

Funding:

Eugene Tang is supported by a NIHR Doctoral Research Fellowship (DRF-2015-08-006).

Louise Robinson is supported by a National Institute for Health Research professorship (NIHR-RP-011-043) and a NIHR Senior Investigator award (NF-SI-0616-10054).

Consent for Publication:

All participants in the study have provided informed written consent. No identifiable personal information has been used.

Competing Interests:

For peer review only

LR reports grants from NIHR Professorship award, grants from NIHR Senior Investigator award, outside the submitted work. The remaining authors declare that they have no competing interests.

Disclaimer:

This paper presents independent research funded by the National Institute for Health Research (NIHR). The views expressed are those of the author(s) and not necessarily those of the NHS, the NIHR or the Department of Health.

Data Sharing Statement:

No further data will be made available.

For peer review only

References

1. Prince M, Wimo W, Guerchet M, et al. World Alzheimer Report 2015: The Global Impact of Dementia, An Analysis of Prevalence, Incidence, Cost and Trends. 2015.
2. Savva GM, Stephan BC, Alzheimer's Society Vascular Dementia Systematic Review G. Epidemiological studies of the effect of stroke on incident dementia: a systematic review. *Stroke* 2010;41(1):e41-6. doi: http://dx.doi.org/10.1161/STROKEAHA.109.559880
3. Pendlebury ST, Rothwell PM. Prevalence, incidence, and factors associated with pre-stroke and post-stroke dementia: a systematic review and meta-analysis. *Lancet Neurol* 2009;8(11):1006 - 18.
4. Douiri A, Rudd AG, Wolfe CD. Prevalence of poststroke cognitive impairment: South London Stroke Register 1995-2010. *Stroke* 2013;44(1):138-45. doi: http://dx.doi.org/10.1161/STROKEAHA.112.670844
5. Kuzma E, Lourida I, Moore SF, et al. Stroke and dementia risk: A systematic review and meta-analysis. *Alzheimer's & dementia : the journal of the Alzheimer's Association* 2018 doi: 10.1016/j.jalz.2018.06.3061 [published Online First: 2018/09/05]
6. Feigin VL, Forouzanfar MH, Krishnamurthi R, et al. Global and regional burden of stroke during 1990-2010: findings from the Global Burden of Disease Study 2010. *Lancet* 2014;383(9913):245-54. [published Online First: 2014/01/23]
7. Prince M, Ali GC, Guerchet M, et al. Recent global trends in the prevalence and incidence of dementia, and survival with dementia. *Alzheimer's research & therapy* 2016;8(1):23. doi: 10.1186/s13195-016-0188-8 [published Online First: 2016/07/31]
8. Baumgart M, Snyder HM, Carrillo MC, et al. Summary of the evidence on modifiable risk factors for cognitive decline and dementia: A population-based perspective. *Alzheimer's & dementia : the journal of the Alzheimer's Association* 2015;11(6):718-26. doi: 10.1016/j.jalz.2015.05.016 [published Online First: 2015/06/06]
9. Stephan BCM, Kurth T, Matthews FE, et al. Dementia risk prediction in the population: are screening models accurate? *Nat Rev Neurol* 2010;6(6):318 - 26.
10. Tang EY, Harrison SL, Errington L, et al. Current Developments in Dementia Risk Prediction Modelling: An Updated Systematic Review. *PloS one* 2015;10(9):e0136181. doi: 10.1371/journal.pone.0136181 [published Online First: 2015/09/04]
11. Kandiah N, Chander RJ, Lin X, et al. Cognitive Impairment after Mild Stroke: Development and Validation of the SIGNAL2 Risk Score. *Journal of Alzheimer's disease : JAD* 2016;49(4):1169-77. doi: 10.3233/jad-150736 [published Online First: 2015/11/26]
12. Lin JH, Lin RT, Tai CT, et al. Prediction of poststroke dementia. *Neurology* 2003;61(3):343-8. [published Online First: 2003/08/13]
13. Stephan BC, Minett T, Muniz Terrera G, et al. Dementia prediction for people with stroke in populations: is mild cognitive impairment a useful concept? *Age and ageing* 2014 doi: 10.1093/ageing/afu085 [published Online First: 2014/07/09]
14. Chander RJ, Lam BYK, Lin X, et al. Development and validation of a risk score (CHANGE) for cognitive impairment after ischemic stroke. *Scientific reports* 2017;7(1):12441. doi: 10.1038/s41598-017-12755-z [published Online First: 2017/10/01]
15. Siontis GC, Tzoulaki I, Siontis KC, et al. Comparisons of established risk prediction models for cardiovascular disease: systematic review. *BMJ (Clinical research ed)* 2012;344:e3318.
16. Johnston SC, Rothwell PM, Nguyen-Huynh MN, et al. Validation and refinement of scores to predict very early stroke risk after transient ischaemic attack. *Lancet* 2007;369(9558):283-92. doi: 10.1016/s0140-6736(07)60150-0 [published Online First: 2007/01/30]
17. Intercollegiate Stroke Working Party. National Clinical Guideline for Stroke. 2016 [published Online First: Fifth Edition]

For peer review only

For peer review only - http://bmjopen.bmj.com/site/about/guidelines.xhtml

18. Tang EYH, Price C, Stephan BCM, et al. Gaps in care for patients with memory deficits after stroke: views of healthcare providers. *BMC health services research* 2017;17(1):634. doi: 10.1186/s12913-017-2569-5 [published Online First: 2017/09/10]
19. Tang EYH, Price C, Stephan BCM, et al. Post-stroke memory deficits and barriers to seeking help: views of patients and carers. *Fam Pract* 2018(In Publication)
20. Braun V, Clarke V. Using thematic analysis in psychology. *Qualitative Research in Psychology* 2006;3(2):77-101. doi: 10.1191/1478088706qp063oa
21. Glaser B. The constant comparison method of qualitative analysis. *Soc Probl* 1965;12:436-45.
22. Legard R, Keegan J, Ward K. In-depth interviews. In: Ritchie J, Lewis J, editors. Qualitative Research Practice: A Guide for Social Science Students and Researchers. London: Sage 2003:pp. 139–169.
23. Saunders B, Sim J, Kingstone T, et al. Saturation in qualitative research: exploring its conceptualization and operationalization. *Quality & quantity* 2018;52(4):1893-907. doi: 10.1007/s11135-017-0574-8 [published Online First: 2018/06/26]
24. O'Brien BC, Harris IB, Beckman TJ, et al. Standards for reporting qualitative research: a synthesis of recommendations. *Academic medicine : journal of the Association of American Medical Colleges* 2014;89(9):1245-51. doi: 10.1097/acm.0000000000000388 [published Online First: 2014/07/01]
25. Tang EY, Birdi R, Robinson L. Attitudes to diagnosis and management in dementia care: views of future general practitioners. *International psychogeriatrics* 2016:1-6. doi: 10.1017/s1041610216001204 [published Online First: 2016/08/10]
26. Koch T, Iliffe S. Rapid appraisal of barriers to the diagnosis and management of patients with dementia in primary care: a systematic review. *BMC family practice* 2010;11:52. doi: 10.1186/1471-2296-11-52 [published Online First: 2010/07/03]
27. Chithiramohan A, Iliffe S, Khattak I. Identifying barriers to diagnosing dementia following incentivisation and policy pressures: General practitioners' perspectives. *Dementia (London, England)* 2016 doi: 10.1177/1471301216682625 [published Online First: 2016/12/13]
28. Hippisley-Cox J, Coupland C, Brindle P. Development and validation of QRISK3 risk prediction algorithms to estimate future risk of cardiovascular disease: prospective cohort study. *BMJ (Clinical research ed)* 2017;357:j2099. doi: 10.1136/bmj.j2099 [published Online First: 2017/05/26]
29. Barfoed BL, Jarbol DE, Paulsen MS, et al. GPs' Perceptions of Cardiovascular Risk and Views on Patient Compliance: A Qualitative Interview Study. *International journal of family medicine* 2015;2015:214146. doi: 10.1155/2015/214146 [published Online First: 2015/10/27]
30. Matz K, Teuschl Y, Firlinger B, et al. Multidomain Lifestyle Interventions for the Prevention of Cognitive Decline After Ischemic Stroke: Randomized Trial. *Stroke* 2015;46(10):2874-80. doi: 10.1161/strokeaha.115.009992 [published Online First: 2015/09/17]
31. Bath PM, Scutt P, Blackburn DJ, et al. Intensive versus Guideline Blood Pressure and Lipid Lowering in Patients with Previous Stroke: Main Results from the Pilot 'Prevention of Decline in Cognition after Stroke Trial' (PODCAST) Randomised Controlled Trial. *PloS one* 2017;12(1):e0164608. doi: 10.1371/journal.pone.0164608 [published Online First: 2017/01/18]
32. Moyer VA. Screening for cognitive impairment in older adults: U.S. Preventive Services Task Force recommendation statement. *Annals of internal medicine* 2014;160(11):791-7. doi: 10.7326/m14-0496 [published Online First: 2014/03/26]
33. Justiss MD, Boustani M, Fox C, et al. Patients' attitudes of dementia screening across the Atlantic. *International journal of geriatric psychiatry* 2009;24(6):632-7. doi: 10.1002/gps.2173 [published Online First: 2008/12/31]
34. Langa KM. Is the risk of Alzheimer's disease and dementia declining? *Alzheimer's research & therapy* 2015;7(1):34. doi: 10.1186/s13195-015-0118-1 [published Online First: 2015/03/31]

For peer review only - http://bmjopen.bmj.com/site/about/guidelines.xhtml

35. Larson EB, Yaffe K, Langa KM. New insights into the dementia epidemic. *The New England journal of medicine* 2013;369(24):2275-7. doi: 10.1056/NEJMp1311405 [published Online First: 2013/11/29]
36. Prince M, Albanese E, Guerchet M, et al. Dementia and Risk Reduction: An Analysis of Protective and Modifiable Factors. 2014.
37. Norton S, Matthews FE, Barnes DE, et al. Potential for primary prevention of Alzheimer's disease: an analysis of population-based data. *Lancet Neurol* 2014;13(8):788-94. doi: 10.1016/s1474-4422(14)70136-x [published Online First: 2014/07/18]
38. Stroke Association. State of the nation: stroke statistics. 2017. https://www.stroke.org.uk/resources/state-nation-stroke-statistics (accessed 14th August 2017).
39. Faircloth CA, Boylstein C, Rittman M, et al. Sudden illness and biographical flow in narratives of stroke recovery. *Sociology of health & illness* 2004;26(2):242-61. doi: 10.1111/j.1467-9566.2004.00388.x [published Online First: 2004/03/19]
40. Campbell S, Manthorpe J, Samsi K, et al. Living with uncertainty: Mapping the transition from pre-diagnosis to a diagnosis of dementia. *Journal of aging studies* 2016;37:40-7. doi: 10.1016/j.jaging.2016.03.001 [published Online First: 2016/05/01]
41. National Institute for Health and Care Excellence. Dementia: assessment, management and support for people living with dementia and their carers 2018. https://www.nice.org.uk/guidance/ng97.
42. Phillips J, Pond CD, Paterson NE, et al. Difficulties in disclosing the diagnosis of dementia: a qualitative study in general practice. *The British journal of general practice : the journal of the Royal College of General Practitioners* 2012;62(601):e546-53. doi: 10.3399/bjgp12X653598 [published Online First: 2012/08/08]
43. Chew-Graham CA, May CR, Perry MS. Qualitative research and the problem of judgement: lessons from interviewing fellow professionals. *Fam Pract* 2002;19(3):285-9. [published Online First: 2002/04/30]

For peer review only

For peer review only - http://bmjopen.bmj.com/site/about/guidelines.xhtml

Table 1. Interview Participants (Patients and Carers)

Unique Identifier (Patients and Carers)	Role	Gender	Age	Follow-up Interview Conducted
P1	Stroke-survivor	Female	80	No
P2	Stroke-survivor	Female	76	Yes
P3	Stroke-survivor	Female	72	Yes
P4	Stroke-survivor	Male	75	Yes
P5	Stroke-survivor	Male	80	Yes
P6	Stroke-survivor	Male	74	Yes
P7	Stroke-survivor	Female	73	Yes
P8	Stroke-survivor	Female	82	Yes
P9	Stroke-survivor	Male	84	No
P10	Stroke-survivor	Male	79	Yes
C1	Carer of P1 (Husband)	Male	79	No
C2	Carer of P4 (Wife)	Female	79	Yes
C3	Carer of P5 (Daughter)	Female	57	Yes
C4	Carer of P6 (Wife)	Female	71	Yes

For peer review only

C5	Carer of P8<br>(Daughter)	Female	60	Yes

For peer review only

Table 2. Interview Participants (Clinicians)

Unique Identifier (Clinicians)	Role	Gender
SC1	Stroke Consultant	Female
SC2	Stroke Specialist Nurse	Female
SC3	Stroke Consultant	Female
SC4	Stroke Consultant	Male
SC5	Stroke Specialist Nurse	Female
SC6	Stroke Physiotherapist (Rehabilitation)	Female
SC7	Stroke Physiotherapist (Acute Care)	Female
SC8	Stroke Occupational Therapist (Acute Care)	Male
SC9	Stroke Occupational Therapist (Rehabilitation)	Female
PC1	General Practitioner with Specialist Interest in Dementia	Male
PC2	General Practitioner	Male
PC3	General Practitioner	Female
PC4	Nurse Practitioner in primary care	Female
PC5	General Practitioner	Female

For peer review only

PC6	Practice Nurse	Female
PC7	Nurse Practitioner in primary care	Female
PC8	General Practitioner	Female

For peer review only

Supplementary Table 1

Standards for Reporting Qualitative Research Checklist[1]

No.	Topic	Item	Page(s)
	**Title and abstract**		
S1	Title	Concise description of the nature and topic of the study Identifying the study as qualitative or indicating the approach (e.g., ethnography, grounded theory) or data collection methods (e.g., interview, focus group) is recommended	1
S2	Abstract	Summary of key elements of the study using the abstract format of the intended publication; typically includes background, purpose, methods, results, and conclusions	2 - 3
	**Introduction**		
S3	Problem formulation	Description and significance of the problem/phenomenon studied; review of relevant theory and empirical work; problem statement	5 – 6
S4	Purpose or research question	Purpose of the study and specific objectives or questions	6
	**Methods**		
S5	Qualitative approach and research paradigm	Qualitative approach (e.g., ethnography, grounded theory, case study, phenomenology, narrative research) and guiding theory if appropriate; identifying the research paradigm (e.g., postpositivist, constructivist/ interpretivist) is also recommended; rationale[b]	8 - 9
S6	Researcher characteristics and reflexivity	Researchers' characteristics that may influence the research, including personal attributes, qualifications/experience, relationship with participants, assumptions, and/or presuppositions; potential or actual interaction between researchers' characteristics and the research questions, approach, methods, results, and/or transferability	7
S7	Context	Setting/site and salient contextual factors; rationale[b]	6 – 9
S8	Sampling strategy	How and why research participants, documents, or events were selected; criteria for deciding when no further sampling was necessary (e.g., sampling saturation); rationale[b]	7 – 9
S9	Ethical issues pertaining to human subjects	Documentation of approval by an appropriate ethics review board and participant consent, or explanation for lack thereof; other confidentiality and data security issues	6 - 7
S10	Data collection methods	Types of data collected; details of data collection procedures including (as appropriate) start and stop dates of data collection and analysis, iterative process, triangulation of sources/methods, and modification of procedures in response to evolving study findings; rationale[b]	8
S11	Data collection instruments and technologies	Description of instruments (e.g., interview guides, questionnaires) and devices (e.g., audio recorders) used for data collection; if/how the instrument(s) changed over the course of the study	8 – 9
S12	Units of study	Number and relevant characteristics of participants, documents, or events included in the study; level of participation (could be reported in results)	31 – 34
S13	Data processing	Methods for processing data prior to and during analysis, including transcription, data entry, data management and security, verification of data integrity, data coding, and anonymization/deidentification of excerpts	8 – 9
S14	Data analysis	Process by which inferences, themes, etc., were identified and developed, including the researchers involved in data analysis; usually references a specific paradigm or approach; rationale [b]	9
S15	Techniques to enhance trustworthiness	Techniques to enhance trustworthiness and credibility of data analysis (e.g., member checking, audit trail, triangulation); rationale[b]	9
	**Results/findings**		
S16	Synthesis and interpretation	Main findings (e.g., interpretations, inferences, and themes); might include development of a theory or model, or integration with prior research or theory	9 – 20
S17	Links to empirical data	Evidence (e.g., quotes, field notes, text excerpts, photographs) to substantiate analytic findings	9 – 20
	**Discussion**		
S18	Integration with prior work, implications, transferability, and contribution(s) to the field	Short summary of main findings; explanation of how findings and conclusions connect to, support, elaborate on, or challenge conclusions of earlier scholarship; discussion of scope of application/ generalizability; identification of unique contribution(s) to scholarship in a discipline or field	20 – 24
S19	Limitations	Trustworthiness and limitations of findings	24
	**Other**		
S20	Conflicts of interest	Potential sources of influence or perceived influence on study conduct and conclusions; how these were managed	26 – 27
S21	Funding	Sources of funding and other support; role of funders in data collection, interpretation, and reporting	26

Reference

1. O'Brien BC, Harris IB, Beckman TJ, et al. Standards for reporting qualitative research: a synthesis of recommendations. *Academic medicine : journal of the Association of American Medical Colleges* 2014;89(9):1245-51. doi: 10.1097/acm.0000000000000388 [published Online First: 2014/07/01]

BMJ Open

The Views of Public and Clinician Stakeholders on Risk Assessment Tools for Post-Stroke Dementia: A Qualitative Study

Journal:	*BMJ Open*
Manuscript ID	bmjopen-2018-025586.R2
Article Type:	Research
Date Submitted by the Author:	25-Jan-2019
Complete List of Authors:	Tang, Eugene; Newcastle University, Institute of Health and Society<br>Exley, Catherine; Newcastle University, Institute of Health and Society<br>Price, Christopher; Newcastle University, Institute of Neuroscience, Stroke Research Group<br>Stephan, Blossom; Newcastle University, Institute of Health and Society<br>Robinson, Louise; Newcastle University, Institute of Health and Society
**Primary Subject Heading**:	Qualitative research
Secondary Subject Heading:	Mental health, Neurology, General practice / Family practice
Keywords:	PRIMARY CARE, STROKE MEDICINE, QUALITATIVE RESEARCH, Dementia < NEUROLOGY

SCHOLARONE™
Manuscripts

The Views of Public and Clinician Stakeholders on Risk Assessment Tools for Post-Stroke Dementia: A Qualitative Study

Dr. Eugene Yee Hing Tang[1,2] e.y.h.tang@newcastle.ac.uk

Prof. Catherine Exley[1] catherine.exley@newcastle.ac.uk

Dr. Christopher Price[3] c.i.m.price@newcastle.ac.uk

Dr. Blossom CM Stephan[1,2] blossom.stephan@newcastle.ac.uk

Prof. Louise Robinson[1,2] a.l.robinson@newcastle.ac.uk

[1] Institute of Health and Society, Newcastle University, Baddiley-Clark, Richardson Road, Newcastle upon Tyne, UK, NE2 4AX

[2] Newcastle University Institute of Ageing, Newcastle University, Campus for Ageing and Vitality, Newcastle upon Tyne, UK, NE4 5PL

[3] Institute of Neuroscience, Stroke Research Group, Newcastle University. Newcastle upon Tyne, UK

Corresponding author: Dr. Eugene Yee Hing Tang, Institute of Health & Society, Newcastle University, Level 2, Newcastle Biomedical Research Building, Campus for Ageing and Vitality, Newcastle upon Tyne, NE4 5PL, T: 0191 208 8758, E: e.y.h.tang@newcastle.ac.uk

For peer review only

Abstract

OBJECTIVE: Stroke-survivors are at increased risk of future dementia. Assessment to identify those at high risk of developing a disease using predictive scores has been utilised in different areas of medicine. A number of risk assessment scores for dementia have been developed but none has been recommended for use clinically. The aim of this qualitative study was to assess the acceptability and feasibility of using a risk assessment tool to predict post-stroke dementia.

DESIGN: Qualitative semi-structured interviews were conducted and analysed thematically. Patients and carers were offered interviews at around 6 (baseline) and 12 (follow-up) months post-stroke; Clinicians were interviewed once.

SETTING: The study was conducted in the North-East of England with stroke patients, family carers and healthcare professionals in primary and secondary care.

PARTICIPANTS: Thirty-nine interviews were conducted (17 clinicians and 15 stroke patients and their carers at baseline. Twelve stroke patients and their carers were interviewed at follow-up; some interviews were conducted in pairs).

RESULTS: Barriers and facilitators to risk assessment were discussed. For patients and carers the focus for facilitators were based on the outcomes of risk assessment e.g. assistance with preparation, diagnosis and for reassurance. For clinicians, facilitators were focused on the process i.e. familiarity in primary care, resource availability in secondary care and collaborative care. For barriers, both groups focussed on the outcome including e.g. the anxiety generated from a potential diagnosis of dementia. For patients/carers a further barrier included concerns about how it may affect their recovery. For clinicians there were concerns about limited interventions and how it would be different from standard care.

For peer review only

CONCLUSIONS: Risk assessment for dementia post-stroke presents challenges given the ramifications of a potential diagnosis of dementia. Attention needs to be given to how information is communicated, and strategies developed to support patients and carers if risk assessment is used.

For peer review only

Strengths and Limitations of the Study:

- To the best of our knowledge this is the first qualitative study to examine critically the views of stroke patients and their family carers and clinicians about the acceptability and feasibility of a risk assessment approach to assist in earlier identification of post-stroke dementia.
- Understanding stakeholder views on risk assessment for dementia can help inform future strategies if risk assessment for dementia is used to assist with earlier diagnosis.
- Patient participants came from one area of England who were able to attend hospital outpatient departments and so may not represent the views and experiences of those with more severe post-stroke sequelae.
- Clinician participants came from one area of England and so may not represent the views of other service models in other regions of the UK.
- It is recognized that clinicians tended to be more familiar with the process of risk assessment and could elaborate further on the process involved.

For peer review only

Introduction

There is currently no cure for dementia and it is estimated that the worldwide economic burden will rise to US$2 trillion by 2030[1]. It has been suggested that the most powerful way to affect costs is by reducing the numbers of people who develop the illness. This may be facilitated by prediction of individual risk for the disease. Stroke is associated with an increased risk of dementia and cognitive impairment[2-4]. A recent meta-analysis found that stroke is a strong independent risk factor for dementia[5]. Stroke incidence and numbers of stroke-survivors are likely to increase due to simultaneous ageing populations and declining stroke mortality rates[6]. Given that the incidence of dementia increases exponentially with age[1 7], this will mean that post-stroke dementia will also become increasingly prevalent. It will therefore be important to identify those at greatest risk of developing dementia following stroke in order to implement strategies to reduce risk. In general, strategies to reduce risk of dementia may include management of cardiovascular risk factors e.g. smoking, diabetes as well as regular physical activity[8].

Risk prediction models for dementia to identify those at higher risk have been developed in whole populations[9 10] with some models specifically developed to predict cognitive impairment and dementia in stroke populations[11-14]. These stroke-specific models predict dementia or cognitive impairment over a relatively short time period (up to 18 months[14]). In spite of the expanding research in this field, none of the dementia risk prediction tools have been clinically implemented. Further, no studies have assessed the feasibility or acceptability of implementing such a strategy in a stroke population. Although risk models are currently used in everyday clinical practice in other branches of medicine, in particular prevention of cardiovascular[15] and cerebrovascular[16] disease, it is unclear how clinicians would feel about using a

For peer review only - http://bmjopen.bmj.com/site/about/guidelines.xhtml

similar strategy to predict dementia, particularly given the stigma surrounding the diagnosis and perceived limited interventions and increased awareness of cognitive difficulties that patients and carers may have following stroke. Further, no studies have evaluated whether using risk assessment tools for dementia would be acceptable to stroke patients themselves.

This paper presents findings from a qualitative study conducted with patients, carers and clinicians, which sought to critically examine their views about the acceptability and feasibility of using risk prediction models in post-stroke care to identify those at greatest risk of future dementia.

Methods

Patient and Public Involvement (PPI)

Patients and members of the public have been involved in the development of this study from the beginning of the proposal. A participant advisory group also oversees the work conducted and annual face-to-face meetings are held to inform them of the study findings. The participant advisory group consists of members from a stroke research patient and carer panel, an organisation aimed at capturing public views about research and from a dementia and neurodegeneration specialty PPI group. The same group reviewed the study materials to ensure suitability particularly for stroke-survivors and their family carers.

Ethical Approval

For peer review only - http://bmjopen.bmj.com/site/about/guidelines.xhtml

The study was conducted in the North East of England. Ethical Approval was obtained from the London – Hampstead Research Ethics Committee (reference 16/LO/0133). Participants provided informed written consent prior to the interview.

Patient and Carer Sampling

Patients and carers were purposively sampled from stroke clinics i.e. to ensure a mix of genders and a range carers were recruited. As part of routine clinical practice in United Kingdom (UK) stroke services, all stroke-survivors are invited to a specialist review at six months after the event which includes a general enquiry about memory concerns[17]. If the patient reported any subjective memory concerns at the clinic and was over the age of 60 and were able to communicate effectively in English, the stroke specialist nurse would provide further study information. Family carers were also recruited if they were involved in the stroke-survivor's care, for example, if they attended the clinic appointment with them. If potential participants were interested in taking part in the study, their details were passed onto the research team. On receipt of this information one researcher (EYHT) would make contact with the patient or carer. He would provide detailed information and an opportunity to ask questions about the study. Following their agreement to participate in the study, participants were asked to take part in an interview immediately following their six-month review and/or around six months later.

Clinician Sampling

General Practitioners (GPs) and secondary care clinicians (e.g. stroke consultants, specialist nurses, physiotherapists and occupational therapists) in the North East of England were contacted to participate in the study. Participants were given an opportunity to ask further questions. Clinicians were purposively sampled to ensure

For peer review only - http://bmjopen.bmj.com/site/about/guidelines.xhtml

that a broad range of care professionals in both primary and secondary care were recruited.

Data collection

Interviews were conducted between April 2016 and August 2017 by one researcher (EYHT) who is a medical doctor. The topic guide was initially derived from relevant literature and expert clinical views within the research team. It was designed to be iterative to enable any topics, which had not been previously identified, to be pursued in subsequent interviews. Face to face semi-structured interviews were conducted with all but one participant (clinician) who had a telephone interview. The patient and family carer were interviewed individually or in pairs as requested by participants. Clinicians were interviewed individually. The part of the interviews focussing on risk assessment asked participants for their views on using risk assessment to help identify stroke-survivors who are most at risk of dementia in the future. They were also asked about the benefits and problems associated with the delivery of this assessment (e.g. who and where it should be carried out), what variables could be used and how best to manage the outcome if individuals were found to be at high or low risk. At follow-up interviews, patient and carer participants were asked to elaborate again on their views of a risk assessment process. Alongside this, the interviews also sought the views of stakeholders on the care experience of post-stroke individuals with memory problems from clinicians, patients and carers. The interviews also looked to understand the impact of post-stroke memory problems on patients and carers. These views on care experience from clinicians[18] and patients and carers[19] have been reported elsewhere. The impact of post-stroke memory problems on patients and carers will be reported separately. This paper reports the views of clinicians, patients and carers on risk assessment

For peer review only

only. The process of risk assessment was described to participants. This was further emphasised with examples of published tools in order to highlight examples of variables used to ensure participant understanding of the process. Informed written consent was obtained from all participants prior to the interview commencing. All interviews were audio-recorded and then transcribed verbatim. To protect participant anonymity, unique identifiers were used throughout the process with identifiable personal data removed.

Data analysis

Interview data was analysed using thematic analysis[20] following the principles of the constant comparative method[21], an iterative approach which allows for issues raised in earlier interviews to be explored subsequently. Data analysis was both deductive and inductive in that we applied learning from previous research and compared with our own data as well as inductively deriving new themes from our data. We ceased data collection when the researcher felt that data saturation occurred. This was defined as being when a full understanding of the participant's perspective[22] and also "informational redundancy" had been reached[23]. One researcher (EYHT) familiarised himself with the dataset and subsequently coded the transcripts line-by-line. Initially, a small subset of transcripts were analysed to identify initial themes and these were discussed between CE and EYHT. Data collection and analysis was iterative and as interviews progressed, further analysis led to new themes emerging and refinement of existing themes and subthemes, which were subsequently grouped into broad categories to facilitate interpretation. The wider team (EYHT, CE, LR, BS and CP) discussed and agreed on the final categories which are presented below. For patient and carer interviews, where follow-up interview data was also obtained, these were analysed as separate interviews to assess for any change in views over time. Data

For peer review only

analysis continued after fieldwork had ceased. There was particular focus to understand what was important to patients, carers and clinicians. Data analysis was facilitated by a data software handing package (NVivo version 11). The paper conforms to the Standards for Reporting Qualitative Research checklist[24] (please see supplementary table 1).

Results

In total, 30 baseline (6 month) interviews were conducted, analysed and compared including: 15 patient and carer interviews (see table 1) and 17 primary and secondary care clinician interviews (see table 2). Two pairs of participants were interviewed together at baseline. Eight stroke-survivors and four carers agreed to a further follow-up interview six months later with nine interviews completed. Three pairs of participants were interviewed together at follow-up. One stroke-survivor declined further follow-up, another stroke-survivor and carer were not followed up due to medical reasons. The data from this study suggest that in terms of risk assessment facilitators and barriers exist to implementation. Whereas patient facilitators focussed on the outcome of the risk assessment, clinicians focussed more on the process of risk assessment for facilitators. Both groups discussed some potential barriers associated with risk assessment focussing on the outcome.

Patient and Carer Views: Facilitators to Risk Assessment Focuses on the Outcome of Assessment

When stroke-survivors and carers discussed the concept of risk assessment, the overarching theme was that an assessment outcome was what was important,

For peer review only - http://bmjopen.bmj.com/site/about/guidelines.xhtml

irrespective of the process and clinicians involved. Participants focussed on several areas of why the outcome was important to them.

For Preparation

Some stroke-survivors were generally positive about receiving a risk assessment for dementia. One stroke-survivor acknowledged that a diagnosis was something that could enable individuals to prepare themselves both at baseline and subsequently at follow-up interview:

"It's the same as knowing and not knowing, if you know that something is approaching. Not everybody is the same with the problem. You might be able to deal with it in a different way or the person supporting you, the nurse or whoever, might be able to find a different way or a more positive way of managing it." (P6, male, stroke-survivor at follow-up interview)

Similarly, for carers, there was the emphasis on what could be done following the assessment. One carer emphasized the importance of looking after the whole person, and, how earlier recognition of a potential dementia diagnosis could ensure strategies were in place to help the individual:

"But I think, if you look at the whole thing of this care of this person, if we knew earlier that you know the chances are that your memory is going to get bad and you are going to go into dementia or whatever, then we can start thinking, "Right, well let's prop it up, let's think of ways in helping your memory as it is, to maintain the level it is before you've got no choice, it's going to get worse." You know, maintaining what you've got and different ways of maintaining it, I think that would help." (C5, female carer (daughter) of stroke-survivor)

For Timely Diagnosis

For peer review only

For some stroke-survivors it did not matter who was performing the risk assessment for dementia or where it was undertaken. What was important was that the diagnosis was reached at the right time:

"I wouldn't say it matters, as long as it's diagnosed at the right time." (P5, male stroke-survivor)

When discussing who should perform the risk assessment, carer participants felt that primary care and the community were regarded as being optimal because of the existing GP-patient relationship. This is because the GP has an overall view of the individual's care:

"I think if you've got a good relationship with your GP I think it should be that, it should be them. Yeah, because you know you trust them you build up a relationship with them so I think that probably, for me that would be the one." (C4, female carer of stroke-survivor)

<u>*For Reassurance*</u>

When stroke-survivor participants were asked about a structured risk assessment process, a further participant reported that the outcome could also ensure some reassurance, either that their symptoms were not related to a dementia diagnosis or that a diagnosis of dementia would be accompanied by support information:

"I think it's reassurance a lot of reassurance with people. You have to give them that to tell them, that "We are there with you. We're going to be helping you." And thats you know, I think that's a good thing." (P2, female stroke-survivor)

Patient and Carer Views: Barriers to Risk Assessment Focuses on the Outcome of Assessment

For peer review only - http://bmjopen.bmj.com/site/about/guidelines.xhtml

Anxiety around a potential diagnosis of dementia

Some carers commented on how the outcome from risk assessment could generate worry and anxiety because of the potential diagnosis of dementia:

"To be honest, I don't know if it would help somebody saying, "You're like this, you're upset because you're like this now, but we actually think you're going to get much worse." Do you know what I mean?" (C3, female carer (daughter) of stroke-survivor)

This person's opinion did not change when she was followed-up six months later. The participant's focus was again on worrying about what could develop and how not knowing about one's risk would actually be more preferable:

"If you could find out and then say, "Right, we've got this medication, or something, that can help you," maybe. But if they're just going to tell you, and then you've got this hanging over your head, and you're thinking, "When is it going to start?" and then you'd be thinking you'd forget something and you'd think, "Oh, that's it, it's coming", which it would be quite normal if you hadn't had that diagnosis, you'd think, "Well I just forgot something, everybody does that." (C3, female carer (daughter) of stroke-survivor at follow-up interview)

However, one carer felt that despite the worry a potential diagnosis may generate, the benefit of this would be to find strategies to maintain cognitive function:

"I think if you had earlier diagnosis, then you would be sort of prepared before things got difficult to handle, or before problems arise, that would be a very good thing. The disadvantages as you say, alarming the carers or the patients themselves, "I'm going to lose my mind." Because, particularly in the older generation, that's a big worry to them. It is a big worry, it's a big worry to all of us, but to older people particularly." (C5, female carer (daughter) of stroke-survivor)

For peer review only - http://bmjopen.bmj.com/site/about/guidelines.xhtml

Concerns about how it may affect their recovery

Not all stroke-survivors were as keen to engage in risk assessment, as there was emphasis on how this may affect them psychologically particularly when their physical deficits had recovered enough to allow them to return to a more usual routine. Therefore, although diagnosis was felt to be important, whether an individual would like to know was also dependent on their subsequent post-stroke recovery:

"That's difficult you know because I mean if you have an early diagnosis you know and say, well "It's going to happen" you know but at the moment now I seem to be progressing through, I'm driving now, you know I'm going back to meetings and whatever. I wonder whether an early diagnosis would restrict that." (P4, male stroke-survivor)

This was particularly evident when patients were followed up six-months later. One participant had actually changed her view over time. Although she had initially felt positive about the process, she then changed her mind when questioned on the same process at her follow-up interview:

"I think my thinking has gone the other way for knowing about that. I think it's sad. I think it's a sad thing. I really do, I think it's really sad that for people to know that they're going to be at high risk, it's a sad thing for it to happen to people, and I don't think I'd want to be one of the sad people. I think I'd just want to be, potter along and that's it." (P2, female, stroke-survivor at follow-up interview)

At follow-up interviews participants also felt that risk assessment should be an individual choice because of the ramifications of the assessment outcome i.e. a potential diagnosis of dementia. Although clinicians may deem it to be helpful, the

For peer review only

choice to undergo risk assessment needs to be a weighed up, which should negate any calls for it to be made a universally applied process:

"I think, medically speaking, yes. On the other hand, does it give people things to worry about that they wouldn't have worried about if you hadn't done the tests? So, I think it depends really on your personal point of view. Do you want to be, you see I would look on the test as saying, well you're at a low, you've got a low risk so that's great but then if it turned out you'd got a high risk are you going to be more worried and less happy than you were before. It's hard to really balance it, isn't it? (P3, female, stroke-survivor at follow-up interview)

Clinician Views: Facilitators to Risk Assessment Focusses on the Process

Clinicians discussed facilitators to risk assessment in terms of how the process may affect the individual and also how the process could be implemented in the future.

When discussing how to implement this process, both primary and secondary care specialists discussed the advantages associated with hosting this process within their own individual teams.

Process familiarity in Primary Care

For primary care, it was about the fact that risk assessment was already a familiar process but that it needed to be individualised:

"I think it's a good tool. We're quite good at using tools, aren't we, but there's always going to be exceptions to the rules and you've got to individualise what you do with it … But sometimes using a score or a tool is a way into a service." (PC4, nurse practitioner in primary care)

It was also recognised by one General Practitioner (GP) that although there is familiarity with risk assessment in primary care, there needs to be caution that the system is not overwhelmed with such tools:

"I do quite like risk profiling. I think we went a little bit crazy with the risk profiling. And there feels to be a lot of competing risk profiling tools, that we're getting a little bit inundated with at the moment … So I think anything like this, I love, if it can be incorporated and brought on to an individual and needs level - so you can think about caring, identifying risk and needs for an individual - would feel great for me" (PC2, General Practitioner)

Secondary care provides specialist input

Stroke care clinicians discussed the facilitators of risk assessment within a specialist setting. This was based on the fact that they felt a responsibility to ensure that post-stroke sequelae are followed up in their specialist services due to the multidisciplinary element of their standard practice and easier access to services. This was particularly important to ensure information could also be given to patients at a time when they may need it the most:

"I think the six-month review tends to be a period of time when the patient's acute side, acute phase of their care has kind of been established, and this is probably the time when they start to recognise problems. And I think it should be within a stroke MDT (multidisciplinary team), not so much focused on by GP's, as such." (SC2, Stroke specialist nurse)

"Well, you need the right support. You need people that actually understand stroke. So I think it would have to be delivered by stroke healthcare professionals. And I think you get so much information when you're initially an inpatient, I think maybe

For peer review only - http://bmjopen.bmj.com/site/about/guidelines.xhtml

that's not the best place to do it … Yeah, it's a big thing to be told that you might develop dementia in a few years' time, so you need psychologists kind of available for if someone needs counselling as a result of that finding. I think it's tricky." (SC6, Stroke physiotherapist)

Collaborative Care

Primary care clinicians commented that there may be a place for both primary and secondary care to work together in identifying those at risk.

"I think primary care would be a completely reasonable place to do that. I guess it's a conversation that could start at diagnosis, at discharge from hospital, like actually, we know that people who have had a stroke are at higher risk of having dementia, these are the things to be aware of, and you know to start that discussion" (PC8, General Practitioner)

Primary and secondary care clinicians felt that such a shared care pathway needed to be formalized to reduce the risk of individuals falling into gaps in care:

"… even if it was picked up in secondary care it's still going to be primary care where most of the management is occurring. So I think it being identified at the six-month follow-up, but then there being a formal sort of mechanism, in which primary care pick it up and process it, would be fine. (PC3, General Practitioner)

"I don't mind where work is done, provided that it is done in a structured and standardised way. If that be, if that can be in primary care that is really good, because that is the long-term follow-up, long-term support, integrating the community … just as long as it can be delivered in a systematic way, and people don't fall through gaps or get inconsistent care." (SC3, Stroke consultant)

For peer review only - http://bmjopen.bmj.com/site/about/guidelines.xhtml

Further, the process of communication between primary and secondary care could also be used in the diagnostic process. It was felt that repeated assessments could help facilitate diagnosis by identifying trends in symptoms:

“You can measure a trend, can't you, if you're using something and measuring something, you can look at a trend. So if its, depends on the type of tool, I guess. But if you did it at you know at the six months review date and then we did it subsequently a year later in primary care, you would see any changes or decline or improvement. So it's a way of, it’s a way of monitoring a trend on how they're doing, I guess. So I don't, I don’t see any reason why it couldn't be done in both and used across both. I don't think we use enough across both.” (PC4, nurse practitioner in primary care)

Clinician Views: Barriers to Risk Assessment Focusses on the Outcome

Limited Interventions Available

Similar to the perspectives of carers, clinicians recognised the anxiety that a risk assessment process might generate and felt that it should be a personal choice to undertake an assessment because of the perceived lack of intervention:

“Yeah, I think I would, I would have degree of anxiety, especially given that the measures that we’re putting in place are … that we could put in place are largely supportive rather than preventative … I would be less confident that I could be giving my patient advice to say, “Well, if we do this, and we do this, and if we do this and you do that then that might move you into an even smaller risk group.” (PC3, General Practitioner)

“Outside research trials, I'm not convinced that there is a definitive value in doing that yet. You know if we get really overwhelming evidence that it’s amenable to

For peer review only - http://bmjopen.bmj.com/site/about/guidelines.xhtml

intervention so you know there's all the theory about blood pressure, and statins, and all the rest of that, but my reading of the evidence on all of that at the moment is that the jury is out whether it makes a difference to cognitive function. So yeah, I'm not convinced that identifying risk, unless you've got a something you can do about it, is actually sensible." (SC4, Stroke consultant)

<u>*Anxiety around a potential diagnosis of dementia*</u>

In recognising the anxiety that this process may generate, one clinician also commented on the fact that patients may not be willing to engage in conversation over the subject of dementia and care should be taken when discussing a potential diagnosis of dementia.

"I think it's good if we tell them that we're looking through and saying, "Look, you know there could be a problem here." But for every single patient, again, because it's quite a still a – not a taboo subject – but it's still not something that people want to talk about … I don't know whether it would be used on every single 'per', you know what I mean, like, everybody." (SC5, Stroke specialist nurse)

<u>*No Change from Standard Practice*</u>

The majority of clinical participants wanted to know, not only what the outcome of the risk assessment would be, but also the resulting care the patient would receive. As part of current routine clinical care, all stroke survivors are offered annual reviews in order to ensure their vascular risk factors e.g. blood pressure and cholesterol are well controlled. In terms of reducing risk, one primary care physician expressed concerns as to what the benefit would to the individual if risk factor modification was already in place anyway particularly with regards to the emotive side of a potential dementia diagnosis. A secondary care specialist questioned the value when there

For peer review only - http://bmjopen.bmj.com/site/about/guidelines.xhtml

was seemingly limited interventions that could be implemented besides managing their cardiovascular risk:

"I guess you've got to be very clear about what it is that you're going to be doing differently for them. So I can see the value if you use a tool for kind of primary prevention, then you're kind of selecting a group of patients out to do something particular with, but I just wonder what would be different about what you do with a risk assessment tool for people who have already had a stroke, when really you know already that it is all about managing their cardiovascular risk so I'm not sure that you would be doing anything different for them." (PC8, General Practitioner)

"Many people will not know of the association between dementia and stroke and many people would not want to know if they were at risk of dementia and again, if you're identifying somebody at risk of a condition that you can't do anything about, what's the right stage to, to do that? However, many of the things you need to do in terms of people being at risk of dementia are the same of the general cardiovascular. So, I'm not sure that there is anything additional that needs to be done about reducing people's risk for dementia over and above general cardiovascular risk." (SC3, Stroke consultant)

Discussion

Main Findings

This is the first study to explore key stakeholders' - stroke survivors, family carers and primary and secondary care clinicians - views on the use of a risk assessment process to predict future dementia in stroke-survivors. It is clear that some of the participants interviewed believed that risk assessment could be of clinical use but raised concerns about it being mandatory. Clinicians highlighted both

For peer review only - http://bmjopen.bmj.com/site/about/guidelines.xhtml

the benefits of collaborative and individual (i.e. primary or secondary) care if dementia risk assessment for stroke survivors was to be implemented.

Clinician facilitators suggest benefits in either primary or secondary care settings but also in a collaborative model of care between the two. This latter finding echoes recommendations from the UK Intercollegiate Stroke Working Party for a collaborative care model, linking community and specialist care, with the aim of integrated long-term follow-up for those presenting neuropsychological problems[17]. Although both primary and secondary care clinicians could see the benefits of carrying this assessment in their own specialties, patients and carers in this study valued their relationship with their GP. Further, primary care clinicians themselves are familiar with the process of risk assessment. A survey of primary care physician trainees found that they were also keen to implement a dementia risk assessment strategy to assist in earlier identification[25]. However, potential barriers have been identified in previous studies, such as system-related factors (lack of support, time constraints)[26 27] and training in dementia[27], which would need to be addressed. Risk assessment is an objective process requiring specific individual variables e.g. age, gender, education. Such data is readily available in primary care in many countries where electronic medical record systems are in place. Further, GPs are already asked to assess cardiovascular risk as part of routine clinical care[28]. However, some GPs themselves do not like using risk assessment tools particularly as the tools do not provide the support needed in communication[29]. Training in communicating the risk assessment process, particularly in the context of dementia, would be required if this were to be implemented in clinical practice. Further, some models, particularly those developed in stroke populations[11] may also include variables such as complex imaging data, which will only be available in secondary care and may be difficult to

For peer review only - http://bmjopen.bmj.com/site/about/guidelines.xhtml

obtain even in specialist settings. If risk assessment were to be conducted in primary care, then the risk assessment models utilising data which can be accessed in primary care, needs to be externally validated in stroke populations to assess their accuracy.

Clinician participants were concerned about whether risk assessment would actually change standard practice. In a stroke population, it is unclear whether identifying those at risk would achieve any additional benefit from a risk factor modification point of view. This is because stroke-survivors already receive annual community follow-up with particular focus on vascular risk factor modification. However, current evidence suggests that development of post-stroke dementia is more than just about vascular risk and would require a different approach e.g. psychological support, cognitive preservation strategies and additional resources. Results from several trials, assessing whether vascular-based interventions can reduce dementia risk, have been largely disappointing[30 31]. These results suggest that perhaps an individual's risk of post-stroke cognitive impairment and dementia includes risk factors beyond vascular risk. Inflammation following a stroke seems to have both positive and negative effects and whether lowering inflammation can prevent post-stroke dementia will need to be addressed in future trials[32].

Currently population screening for dementia is not recommended due to a lack of evidence evaluating risks and benefits[33], despite positive views from older adults[34]. Risk assessment can target high-risk groups rather than the general population. Recent evidence has found a decline in age-specific incidence of dementia, particularly in high-income countries, suggesting that rising levels of education and modifying cardiovascular risk may have driven a decline in dementia risk[35 36]. Indeed, the importance of modifiable risk factor reduction for dementia was

For peer review only - http://bmjopen.bmj.com/site/about/guidelines.xhtml

reported in the World Alzheimer Report (2014)[37] and around a third of Alzheimer's disease cases worldwide might be attributable to modifiable risk factors[38]. Risk assessment tools utilize these modifiable risk factors to predict risk. Similar to other branches of medicine where risk assessment is utilized to predict risk of a future illness, it would be hoped that this approach could reduce one's risk of future dementia. Stroke affects more than 100,000 people in the UK per year[39], creating a large population with cognitive deficits and/or at high risk of future decline who may benefit from risk assessment for dementia. However, participant groups in this study, particularly clinicians, reported that given the potential ramifications of risk assessment, individuals should be given the choice of whether to undergo assessment. Stroke-survivors were generally positive about such an approach but agreed that it should be up to the individual and the family rather than applied universally. Participants in this study recognised the anxiety this process could generate particularly when the perceived possible interventions for dementia are limited. The National Institute for Health and Care Excellence have recently updated their guidance and have concluded that case finding should only be conducted as part of a clinical trial, which also provides an intervention[40]. Therefore, careful discussion needs to be adopted with the patient and their carers before undertaking such a process in any setting. In the context of the dementia diagnostic journey, transition from living with an undiagnosed memory problem to being diagnosed with a dementia illness is underpinned by uncertainty[41]. Although risk assessment certainly does not provide any certainty for a dementia illness, the discussions and objective evaluation using the tools may help the individual's process their current condition and assist in the preparation for a potential diagnosis of dementia.

For peer review only - http://bmjopen.bmj.com/site/about/guidelines.xhtml

Preparation was mentioned by participants in this study as a facilitator for risk assessment.

Clinical Implications

Case finding for dementia involves actively assessing individuals at risk of a future dementia illness, which at present is only recommended in clinical trial settings due to a lack of post-assessment intervention[42]. Once a suitable intervention is found however, the views of those conducting the assessment and the recipients of such an assessment will need to be assessed. Similarly there will be challenges with regards to assessment of capacity when performing risk assessment for this at-risk population. It is also important to note that GPs find communicating the diagnosis of dementia difficult[43]. Although risk assessment is not providing a diagnosis of dementia, careful consideration will be required in training health professionals in communicating the concept of risk for a disease such as dementia. From this study we have identified the priorities according to each stakeholder group which would need to be addressed prior to clinical implementation in the future.

Limitations

The participants in this study came from one area of England and were Caucasian. Patient participants were also well enough to attend outpatient assessment clinics. Future studies could look to explore views in other populations including views from minority ethnic groups, patients with more severe stroke-related impairments and different service models. Due to familiarity, it is recognized that clinicians expanded more around the risk assessment process. Despite this being the case, patients and carers were given the opportunity to understand the concept of risk assessment as part of the interview process but the emphasis on a need for a diagnosis and good

For peer review only

care was what was important for them. Participants were also aware that the interviewer was also a primary care clinician, which may have the potential to introduce bias into participant responses. This is because a clinician interviewer may be viewed as an expert and judge in clinical decision making and moral judgements made[44]. On the other hand interviews tend to be broader in scope and richer in data when conducted by a clinician researcher [44]. Further, both clinical and non-clinical members contributed to the analysis of the data to minimize the effect this may have had.

Conclusions and Future Research

Timely recognition of those at risk of dementia is crucial to enable individuals early access treatment and support. Although dementia screening after stroke is not yet advocated on preventative grounds, assessing risk has some potential benefits for individuals who make an informed choice to participate. There would need to be better cohesiveness of communication between primary and secondary care, with more support placed in the community. Further, it should be recognised that if risk assessment were to be incorporated into clinical practice, this will potentially place additional burdens on a dementia diagnostic service which is already overstretched. Next steps are to identify which tool to use, how best to manage those who are deemed high-risk individuals and whether there are any interventions, which can reduce their risk. Future studies will need to look specifically at what factors put a stroke-survivor at risk that could be potentially modified and also whether there are specific interventions suitable to a post-stroke population to reduce risk.

For peer review only

Acknowledgements:

The authors would like to thank the participant advisory group for their advice on the study materials used.

List of Abbreviations

**GP**	General Practitioner
**UK**	United Kingdom

<u>Declarations</u>

Author Contributions:

ET conceived the framework for this study. ET collected, analysed and interpreted the data. ET prepared the manuscript for submission.

CE helped to conceive the framework for this study and assisted with the analysis of the data and contributed to the drafting of the manuscript. CE also critically reviewed and edited the manuscript.

CP helped to conceive the framework for this study, assisted with the analysis of the data and critically reviewed and edited the manuscript.

BS helped to conceive the framework for this study, assisted with the analysis of the data and critically reviewed and edited the manuscript.

LR helped to conceive the framework for this study, assisted with the analysis of the data and critically reviewed and edited the manuscript.

Funding:

Eugene Tang is supported by a NIHR Doctoral Research Fellowship (DRF-2015-08-006).

For peer review only

Louise Robinson is supported by a National Institute for Health Research professorship (NIHR-RP-011-043) and a NIHR Senior Investigator award (NF-SI-0616-10054).

Consent for Publication:

All participants in the study have provided informed written consent. No identifiable personal information has been used.

Competing Interests:

LR reports grants from NIHR Professorship award, grants from NIHR Senior Investigator award, outside the submitted work. The remaining authors declare that they have no competing interests.

Disclaimer:

This paper presents independent research funded by the National Institute for Health Research (NIHR). The views expressed are those of the author(s) and not necessarily those of the NHS, the NIHR or the Department of Health.

Data Sharing Statement:

No further data will be made available.

For peer review only

References

1. Prince M, Wimo W, Guerchet M, et al. World Alzheimer Report 2015: The Global Impact of Dementia, An Analysis of Prevalence, Incidence, Cost and Trends. 2015.
2. Savva GM, Stephan BC, Alzheimer's Society Vascular Dementia Systematic Review G. Epidemiological studies of the effect of stroke on incident dementia: a systematic review. *Stroke; a journal of cerebral circulation* 2010;41(1):e41-6. doi: http://dx.doi.org/10.1161/STROKEAHA.109.559880
3. Pendlebury ST, Rothwell PM. Prevalence, incidence, and factors associated with pre-stroke and post-stroke dementia: a systematic review and meta-analysis. *The Lancet Neurology* 2009;8(11):1006 - 18.
4. Douiri A, Rudd AG, Wolfe CD. Prevalence of poststroke cognitive impairment: South London Stroke Register 1995-2010. *Stroke; a journal of cerebral circulation* 2013;44(1):138-45. doi: 10.1161/strokeaha.112.670844 [published Online First: 2012/11/15]
5. Kuzma E, Lourida I, Moore SF, et al. Stroke and dementia risk: A systematic review and meta-analysis. *Alzheimer's & dementia : the journal of the Alzheimer's Association* 2018 doi: 10.1016/j.jalz.2018.06.3061 [published Online First: 2018/09/05]
6. Feigin VL, Forouzanfar MH, Krishnamurthi R, et al. Global and regional burden of stroke during 1990-2010: findings from the Global Burden of Disease Study 2010. *Lancet* 2014;383(9913):245-54. [published Online First: 2014/01/23]
7. Prince M, Ali GC, Guerchet M, et al. Recent global trends in the prevalence and incidence of dementia, and survival with dementia. *Alzheimer's research & therapy* 2016;8(1):23. doi: 10.1186/s13195-016-0188-8 [published Online First: 2016/07/31]
8. Baumgart M, Snyder HM, Carrillo MC, et al. Summary of the evidence on modifiable risk factors for cognitive decline and dementia: A population-based perspective. *Alzheimer's & dementia : the journal of the Alzheimer's Association* 2015;11(6):718-26. doi: 10.1016/j.jalz.2015.05.016 [published Online First: 2015/06/06]
9. Stephan BCM, Kurth T, Matthews FE, et al. Dementia risk prediction in the population: are screening models accurate? *Nat Rev Neurol* 2010;6(6):318 - 26.
10. Tang EY, Harrison SL, Errington L, et al. Current Developments in Dementia Risk Prediction Modelling: An Updated Systematic Review. *PloS one* 2015;10(9):e0136181. doi: 10.1371/journal.pone.0136181 [published Online First: 2015/09/04]
11. Kandiah N, Chander RJ, Lin X, et al. Cognitive Impairment after Mild Stroke: Development and Validation of the SIGNAL2 Risk Score. *Journal of Alzheimer's disease : JAD* 2016;49(4):1169-77. doi: 10.3233/jad-150736 [published Online First: 2015/11/26]
12. Lin JH, Lin RT, Tai CT, et al. Prediction of poststroke dementia. *Neurology* 2003;61(3):343-8. [published Online First: 2003/08/13]
13. Stephan BC, Minett T, Muniz Terrera G, et al. Dementia prediction for people with stroke in populations: is mild cognitive impairment a useful concept? *Age and ageing* 2014 doi: 10.1093/ageing/afu085 [published Online First: 2014/07/09]
14. Chander RJ, Lam BYK, Lin X, et al. Development and validation of a risk score (CHANGE) for cognitive impairment after ischemic stroke. *Scientific reports* 2017;7(1):12441. doi: 10.1038/s41598-017-12755-z [published Online First: 2017/10/01]
15. Siontis GC, Tzoulaki I, Siontis KC, et al. Comparisons of established risk prediction models for cardiovascular disease: systematic review. *BMJ (Clinical research ed)* 2012;344:e3318.
16. Johnston SC, Rothwell PM, Nguyen-Huynh MN, et al. Validation and refinement of scores to predict very early stroke risk after transient ischaemic attack. *Lancet* 2007;369(9558):283-92. doi: 10.1016/s0140-6736(07)60150-0 [published Online First: 2007/01/30]
17. Intercollegiate Stroke Working Party. National Clinical Guideline for Stroke. 2016 [published Online First: Fifth Edition]

For peer review only - http://bmjopen.bmj.com/site/about/guidelines.xhtml

18. Tang EYH, Price C, Stephan BCM, et al. Gaps in care for patients with memory deficits after stroke: views of healthcare providers. *BMC health services research* 2017;17(1):634. doi: 10.1186/s12913-017-2569-5 [published Online First: 2017/09/10]
19. Tang EYH, Price C, Stephan BCM, et al. Post-stroke memory deficits and barriers to seeking help: views of patients and carers. *Fam Pract* 2018(In Publication)
20. Braun V, Clarke V. Using thematic analysis in psychology. *Qualitative Research in Psychology* 2006;3(2):77-101. doi: 10.1191/1478088706qp063oa
21. Glaser B. The constant comparison method of qualitative analysis. *Soc Probl* 1965;12:436-45.
22. Legard R, Keegan J, Ward K. In-depth interviews. In: Ritchie J, Lewis J, editors. Qualitative Research Practice: A Guide for Social Science Students and Researchers. London: Sage 2003:pp. 139–169.
23. Saunders B, Sim J, Kingstone T, et al. Saturation in qualitative research: exploring its conceptualization and operationalization. *Quality & quantity* 2018;52(4):1893-907. doi: 10.1007/s11135-017-0574-8 [published Online First: 2018/06/26]
24. O'Brien BC, Harris IB, Beckman TJ, et al. Standards for reporting qualitative research: a synthesis of recommendations. *Academic medicine : journal of the Association of American Medical Colleges* 2014;89(9):1245-51. doi: 10.1097/acm.0000000000000388 [published Online First: 2014/07/01]
25. Tang EY, Birdi R, Robinson L. Attitudes to diagnosis and management in dementia care: views of future general practitioners. *International psychogeriatrics* 2016:1-6. doi: 10.1017/s1041610216001204 [published Online First: 2016/08/10]
26. Koch T, Iliffe S. Rapid appraisal of barriers to the diagnosis and management of patients with dementia in primary care: a systematic review. *BMC Fam Pract* 2010;11:52. doi: 10.1186/1471-2296-11-52 [published Online First: 2010/07/03]
27. Chithiramohan A, Iliffe S, Khattak I. Identifying barriers to diagnosing dementia following incentivisation and policy pressures: General practitioners' perspectives. *Dementia (London, England)* 2016 doi: 10.1177/1471301216682625 [published Online First: 2016/12/13]
28. Hippisley-Cox J, Coupland C, Brindle P. Development and validation of QRISK3 risk prediction algorithms to estimate future risk of cardiovascular disease: prospective cohort study. *BMJ (Clinical research ed)* 2017;357:j2099. doi: 10.1136/bmj.j2099 [published Online First: 2017/05/26]
29. Barfoed BL, Jarbol DE, Paulsen MS, et al. GPs' Perceptions of Cardiovascular Risk and Views on Patient Compliance: A Qualitative Interview Study. *International journal of family medicine* 2015;2015:214146. doi: 10.1155/2015/214146 [published Online First: 2015/10/27]
30. Matz K, Teuschl Y, Firlinger B, et al. Multidomain Lifestyle Interventions for the Prevention of Cognitive Decline After Ischemic Stroke: Randomized Trial. *Stroke; a journal of cerebral circulation* 2015;46(10):2874-80. doi: 10.1161/strokeaha.115.009992 [published Online First: 2015/09/17]
31. Bath PM, Scutt P, Blackburn DJ, et al. Intensive versus Guideline Blood Pressure and Lipid Lowering in Patients with Previous Stroke: Main Results from the Pilot 'Prevention of Decline in Cognition after Stroke Trial' (PODCAST) Randomised Controlled Trial. *PloS one* 2017;12(1):e0164608. doi: 10.1371/journal.pone.0164608 [published Online First: 2017/01/18]
32. Mijajlovic MD, Pavlovic A, Brainin M, et al. Post-stroke dementia - a comprehensive review. *BMC medicine* 2017;15(1):11. doi: 10.1186/s12916-017-0779-7 [published Online First: 2017/01/18]
33. Moyer VA. Screening for cognitive impairment in older adults: U.S. Preventive Services Task Force recommendation statement. *Annals of internal medicine* 2014;160(11):791-7. doi: 10.7326/m14-0496 [published Online First: 2014/03/26]

For peer review only

34. Justiss MD, Boustani M, Fox C, et al. Patients' attitudes of dementia screening across the Atlantic. *International journal of geriatric psychiatry* 2009;24(6):632-7. doi: 10.1002/gps.2173 [published Online First: 2008/12/31]
35. Langa KM. Is the risk of Alzheimer's disease and dementia declining? *Alzheimer's research & therapy* 2015;7(1):34. doi: 10.1186/s13195-015-0118-1 [published Online First: 2015/03/31]
36. Larson EB, Yaffe K, Langa KM. New insights into the dementia epidemic. *The New England journal of medicine* 2013;369(24):2275-7. doi: 10.1056/NEJMp1311405 [published Online First: 2013/11/29]
37. Prince M, Albanese E, Guerchet M, et al. Dementia and Risk Reduction: An Analysis of Protective and Modifiable Factors. 2014.
38. Norton S, Matthews FE, Barnes DE, et al. Potential for primary prevention of Alzheimer's disease: an analysis of population-based data. *The Lancet Neurology* 2014;13(8):788-94. doi: 10.1016/s1474-4422(14)70136-x [published Online First: 2014/07/18]
39. Stroke Association. State of the nation: stroke statistics. 2017. https://www.stroke.org.uk/resources/state-nation-stroke-statistics (accessed 14th August 2017).
40. Faircloth CA, Boylstein C, Rittman M, et al. Sudden illness and biographical flow in narratives of stroke recovery. *Sociology of health & illness* 2004;26(2):242-61. doi: 10.1111/j.1467-9566.2004.00388.x [published Online First: 2004/03/19]
41. Campbell S, Manthorpe J, Samsi K, et al. Living with uncertainty: Mapping the transition from pre-diagnosis to a diagnosis of dementia. *Journal of aging studies* 2016;37:40-7. doi: 10.1016/j.jaging.2016.03.001 [published Online First: 2016/05/01]
42. National Institute for Health and Care Excellence. Dementia: assessment, management and support for people living with dementia and their carers 2018. https://www.nice.org.uk/guidance/ng97.
43. Phillips J, Pond CD, Paterson NE, et al. Difficulties in disclosing the diagnosis of dementia: a qualitative study in general practice. *The British journal of general practice : the journal of the Royal College of General Practitioners* 2012;62(601):e546-53. doi: 10.3399/bjgp12X653598 [published Online First: 2012/08/08]
44. Chew-Graham CA, May CR, Perry MS. Qualitative research and the problem of judgement: lessons from interviewing fellow professionals. *Fam Pract* 2002;19(3):285-9. [published Online First: 2002/04/30]

For peer review only

Table 1. Interview Participants (Patients and Carers)

Unique Identifier (Patients and Carers)	Role	Gender	Age	Follow-up Interview Conducted
P1	Stroke-survivor	Female	80	No
P2	Stroke-survivor	Female	76	Yes
P3	Stroke-survivor	Female	72	Yes
P4	Stroke-survivor	Male	75	Yes
P5	Stroke-survivor	Male	80	Yes
P6	Stroke-survivor	Male	74	Yes
P7	Stroke-survivor	Female	73	Yes
P8	Stroke-survivor	Female	82	Yes
P9	Stroke-survivor	Male	84	No
P10	Stroke-survivor	Male	79	Yes
C1	Carer of P1 (Husband)	Male	79	No
C2	Carer of P4 (Wife)	Female	79	Yes
C3	Carer of P5 (Daughter)	Female	57	Yes
C4	Carer of P6 (Wife)	Female	71	Yes

For peer review only

C5	Carer of P8 (Daughter)	Female	60	Yes

For peer review only

Table 2. Interview Participants (Clinicians)

Unique Identifier (Clinicians)	Role	Gender
SC1	Stroke Consultant	Female
SC2	Stroke Specialist Nurse	Female
SC3	Stroke Consultant	Female
SC4	Stroke Consultant	Male
SC5	Stroke Specialist Nurse	Female
SC6	Stroke Physiotherapist (Rehabilitation)	Female
SC7	Stroke Physiotherapist (Acute Care)	Female
SC8	Stroke Occupational Therapist (Acute Care)	Male
SC9	Stroke Occupational Therapist (Rehabilitation)	Female
PC1	General Practitioner with Specialist Interest in Dementia	Male
PC2	General Practitioner	Male
PC3	General Practitioner	Female
PC4	Nurse Practitioner in primary care	Female
PC5	General Practitioner	Female

For peer review only

PC6	Practice Nurse	Female
PC7	Nurse Practitioner in primary care	Female
PC8	General Practitioner	Female

For peer review only

Supplementary Table 1

Standards for Reporting Qualitative Research Checklist[1]

No.	Topic	Item	Page(s)
	**Title and abstract**		
S1	Title	Concise description of the nature and topic of the study Identifying the study as qualitative or indicating the approach (e.g., ethnography, grounded theory) or data collection methods (e.g., interview, focus group) is recommended	1
S2	Abstract	Summary of key elements of the study using the abstract format of the intended publication; typically includes background, purpose, methods, results, and conclusions	2 - 3
	**Introduction**		
S3	Problem formulation	Description and significance of the problem/phenomenon studied; review of relevant theory and empirical work; problem statement	5 – 6
S4	Purpose or research question	Purpose of the study and specific objectives or questions	6
	**Methods**		
S5	Qualitative approach and research paradigm	Qualitative approach (e.g., ethnography, grounded theory, case study, phenomenology, narrative research) and guiding theory if appropriate; identifying the research paradigm (e.g., postpositivist, constructivist/ interpretivist) is also recommended; rationale[b]	8 - 9
S6	Researcher characteristics and reflexivity	Researchers' characteristics that may influence the research, including personal attributes, qualifications/experience, relationship with participants, assumptions, and/or presuppositions; potential or actual interaction between researchers' characteristics and the research questions, approach, methods, results, and/or transferability	8
S7	Context	Setting/site and salient contextual factors; rationale[b]	7 – 9
S8	Sampling strategy	How and why research participants, documents, or events were selected; criteria for deciding when no further sampling was necessary (e.g., sampling saturation); rationale[b]	7 – 10
S9	Ethical issues pertaining to human subjects	Documentation of approval by an appropriate ethics review board and participant consent, or explanation for lack thereof; other confidentiality and data security issues	6 - 7
S10	Data collection methods	Types of data collected; details of data collection procedures including (as appropriate) start and stop dates of data collection and analysis, iterative process, triangulation of sources/methods, and modification of procedures in response to evolving study findings; rationale[b]	8 – 9
S11	Data collection instruments and technologies	Description of instruments (e.g., interview guides, questionnaires) and devices (e.g., audio recorders) used for data collection; if/how the instrument(s) changed over the course of the study	8 – 9
S12	Units of study	Number and relevant characteristics of participants, documents, or events included in the study; level of participation (could be reported in results)	31 – 34
S13	Data processing	Methods for processing data prior to and during analysis, including transcription, data entry, data management and security, verification of data integrity, data coding, and anonymization/deidentification of excerpts	8 – 10
S14	Data analysis	Process by which inferences, themes, etc., were identified and developed, including the researchers involved in data analysis; usually references a specific paradigm or approach; rationale [b]	9 – 10
S15	Techniques to enhance trustworthiness	Techniques to enhance trustworthiness and credibility of data analysis (e.g., member checking, audit trail, triangulation); rationale[b]	9 – 10
	**Results/findings**		
S16	Synthesis and interpretation	Main findings (e.g., interpretations, inferences, and themes); might include development of a theory or model, or integration with prior research or theory	10 – 20
S17	Links to empirical data	Evidence (e.g., quotes, field notes, text excerpts, photographs) to substantiate analytic findings	10 – 20
	**Discussion**		
S18	Integration with prior work, implications, transferability, and contribution(s) to the field	Short summary of main findings; explanation of how findings and conclusions connect to, support, elaborate on, or challenge conclusions of earlier scholarship; discussion of scope of application/ generalizability; identification of unique contribution(s) to scholarship in a discipline or field	20 – 24
S19	Limitations	Trustworthiness and limitations of findings	24 – 25
	**Other**		
S20	Conflicts of interest	Potential sources of influence or perceived influence on study conduct and conclusions; how these were managed	27
S21	Funding	Sources of funding and other support; role of funders in data collection, interpretation, and reporting	26 – 27

Reference

1. O'Brien BC, Harris IB, Beckman TJ, et al. Standards for reporting qualitative research: a synthesis of recommendations. *Academic medicine : journal of the Association of American Medical Colleges* 2014;89(9):1245-51. doi: 10.1097/acm.0000000000000388 [published Online First: 2014/07/01]
